# Nowcasting Vector Mosquito Abundance and Determining Its Association With Malaria Epidemics in South Korea

**DOI:** 10.1155/tbed/9959287

**Published:** 2025-01-17

**Authors:** Taehee Chang, Saebom Choi, Hojong Jun, Jong-Yil Chai, Sang Hoon Song, Sehyeon Kim, Joon-Sup Yeom, Sung-il Cho, Kyung-Duk Min

**Affiliations:** ^1^Department of Public Health Sciences, Graduate School of Public Health, Seoul National University, Seoul, Republic of Korea; ^2^Department of Medical Environmental Biology and Tropical Medicine, School of Medicine, Kangwon National University, Chuncheon, Republic of Korea; ^3^Department of Tropical Medicine and Parasitology, Seoul National University College of Medicine, Seoul, Republic of Korea; ^4^Department of Laboratory Medicine, Seoul National University College of Medicine and Hospital, Seoul, Republic of Korea; ^5^Medipeace Peru Office, Medipeace, Lima, Peru; ^6^Department of Internal Medicine, Yonsei University College of Medicine, Seoul, Republic of Korea; ^7^Institute of Health and Environment, Seoul National University, Seoul, Republic of Korea; ^8^College of Veterinary Medicine, Chungbuk National University, Cheongju, Republic of Korea

**Keywords:** early warning system, machine learning model, mosquito abundance, nowcasting, *Plasmodium vivax* malaria

## Abstract

Since a resurgence occurred in 1993, malaria has remained an endemic disease in the Republic of Korea (ROK). A major challenge is the inaccessibility of current vector mosquito abundance data due to a 2-week reporting delay, which limits timely implementation of control measures. We aimed to nowcast mosquito abundance and assess its utility by evaluating the predictive value of mosquito abundance for malaria epidemic peaks. We used machine learning models to nowcast mosquito abundance, employing gradient boosting models (GBMs), extreme gradient boosting (XGB), and an ensemble model combining both. Various meteorological factors served as predictors. The models were trained with data from mosquito collection sites between 2009 and 2021 and tested with data from 2022. To evaluate the utility of nowcasting, we calculated the effective reproduction number (*R*_t_), which can indicate malaria epidemic peaks. Generalized linear models (GLMs) were then used to assess the impact of vector mosquito abundance on *R*_t_. The ensemble models demonstrated the best performance in nowcasting mosquito abundance, with a root mean square error (RMSE) of 0.90 and *R*-squared value (*R*^2^) value of 0.85. The GBM model showed an RMSE of 0.91 and *R*^2^ of 0.84, while the XGB model had an RMSE of 0.92 and *R*^2^ of 0.85. Additionally, the *R*^2^ of the GLMs predicting *R*_t_ using mosquito abundance 2 weeks in advance was >0.72 for all provinces. The mosquito abundance coefficients were also significant. We constructed reliable models to nowcast mosquito abundance. These outcomes could potentially be incorporated into a malaria early warning system. Our study provides evidence to support the development of malaria management strategies in regions where malaria remains a public health challenge.

## 1. Introduction

Malaria is one of the most widespread parasitic diseases worldwide, with approximately 3.2 billion people at risk of infection [1]. Indigenous *Plasmodium vivax* malaria was thought to have been eradicated from the Republic of Korea (ROK) in 1984 [2]. However, a resurgence occurred in 1993 with a case near the demilitarized zone (DMZ) in northern Gyeonggi-do, adjacent to the Democratic People's ROK [3]. After its reemergence, *P. vivax* malaria again became endemic in the ROK, peaking in 2007 with 2192 cases—one of the highest incidence rates among countries with temperate climates [2]. Since 2010, due to effective control strategies, the annual number of reported cases has continuously decreased, ranging between approximately 400 and 800 [4]. Nonetheless, *P. vivax* malaria continues to pose public health challenges in the northern parts of the ROK near the DMZ, and its elimination remains elusive.

Vector surveillance has been conducted to support decision-making regarding the elimination goal; however, its practical utility in influencing disease control policy has not been clearly established. In the ROK, eight species of *Anopheles* mosquitoes have been identified [5], with *Anopheles sinensis* considered the primary vector of *P. vivax* malaria [6]. The numbers of malaria vector mosquitoes and human malaria cases typically begin to rise in early spring and decrease significantly in late autumn, displaying a corresponding trend [3, 7]. However, these trends do not establish a clear relationship between mosquito abundance and malaria incidence. For example, regions with a high density of malaria vector mosquitoes do not necessarily have high malaria incidences [4, 8]. Additionally, the onset of malaria cases often precedes the active period of malaria vectors [4, 8]. Considering that the association between malaria vector mosquitoes and the incidence of malaria is not well-defined, vector control program planning is challenging. Therefore, it is essential to quantitatively assess these associations to establish refined objectives for malaria management and conduct effective control programs.

If associations between vector mosquito abundance and malaria epidemics are well-established, monitoring mosquito populations could be crucial for effective disease control. Since 2009, the Division of Vectors and Parasitic Diseases at the Korea Disease Control and Prevention Agency (KDCA) has conducted surveillance of vector mosquitoes in malaria-risk areas, publishing information on mosquito abundance and *P. vivax* infection rates [8]. However, timely forecast data for malaria epidemics are limited due to an approximate 2-week delay in data collection and processing. Previous studies have suggested that the abundance of Anopheles mosquitoes can be estimated based on various meteorological factors. Studies conducted in the ROK have shown that meteorological factors, such as ambient temperature, humidity, and precipitation, can be used to estimate mosquito distribution [9, 10]. Similar studies conducted in China, another temperate zone, have demonstrated that meteorological factors significantly affect mosquito distribution [11]. Therefore, it is plausible to use these ecological factors to predict vector abundance in real-time, thereby, addressing the reporting delay.

In this study, we aimed to nowcast mosquito abundance at each monitoring site to address the current reporting delays. We applied a “nowcasting” approach, which estimates current mosquito abundance using historical data. Specifically, we developed machine learning models that incorporate mosquito occurrence data (e.g., from 2 weeks and earlier) and real-time meteorological factors to infer the current state of mosquito abundance. Additionally, we demonstrated the utility of nowcasting by showing how accurately vector mosquito abundance can predict the peak of malaria epidemics.

## 2. Methods

### 2.1. Study Design

**Figure 1:**
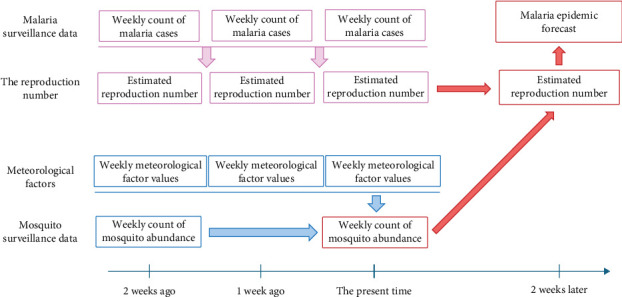
Schematic description of study design. The study design involved inputting meteorological factors and mosquito abundance from 2 weeks prior to the machine learning models to nowcast the current mosquito abundance. This outcome was then used to forecast the *R*_t_ 2 weeks in advance.

**Figure 2:**
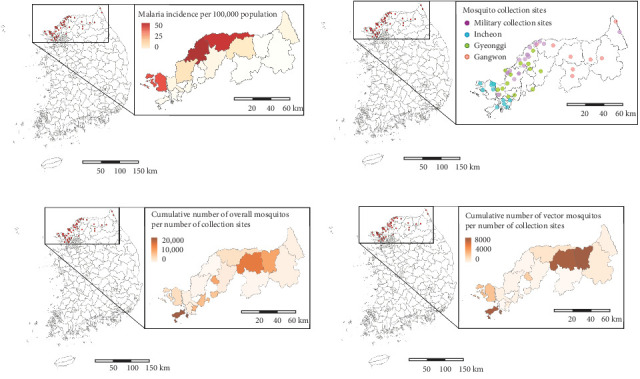
Descriptive maps of study area. (A) Average malaria incidence per 100,000 population in the study area between 2009 and 2022. (B) Location of mosquito collection sites operated by Korea Disease Control and Prevention Agency (KDCA). (C) and (D) display the cumulative number of overall and vector mosquitoes per number of collection sites between 2009 and 2022, respectively.

We nowcasted mosquito abundance using machine learning models and verified whether the results could serve as reliable predictors of malaria epidemic peaks.  Figure 1 illustrates the schematic diagram of the study process. To nowcast mosquito abundance, we utilized meteorological factors combined with mosquito abundance data from previous weeks as explanatory variables. We assessed the peak of malaria cases based on the effective reproduction number, *R*_t_, an important epidemiological measure of the transmission potential of an infectious pathogen [12]. Then, we examined the association between *R*_t_ and mosquito abundance. The study area comprises parts of three provinces (the first administrative level, Si-Do): Incheon-si, Gyeonggi-do, and Gangwon-do, which are designated by the KDCA as high-risk regions for malaria (Figure 2A and Figure S1).

### 2.2. Mosquito Surveillance

Since 2009, the Division of Vectors and Parasitic Diseases at the KDCA has conducted surveys of vector mosquito abundance in high-risk malaria regions, including Incheon, Gyeonggi, and Gangwon [8]. Additionally, since 2019, with the cooperation of military units, surveys have also been conducted at collection points near the DMZ [13]. The number of mosquito collection sites was maintained at 20 from 2009 to 2018, increased to 44 in 2019 and 51 in 2020, and has been maintained at 50 since 2021 (Figure 2B and Table S1). In this study, we utilized mosquito abundance data from 49 observation points, including 35 in civilian areas and 14 in military zones, for consistency. Mosquito collection considered the timing of mosquito activity and regional characteristics. In civilian areas, collections were conducted between April and October (7 months) using black light traps. In military areas near the DMZ, located in mountainous regions with lower temperatures, collections were carried out between May and September (5 months) using light-emitting diode (LED) traps. Mosquitoes collected at each site were identified under a stereomicroscope, and only female mosquitoes were counted.

### 2.3. Meteorological Data

We collected daily meteorological data from nine meteorological stations (Automated Synoptic Observing System, ASOS; Figure S2) [14]. We computed weekly values for these meteorological factors to align with the KDCA's method of calculating weekly mosquito surveillance data and malaria cases. Based on data from each meteorological station, we performed spatial interpolation to assign meteorological values to the mosquito collection sites. We used the kriging method for the interpolation process [15]. The list of computed predictors is presented in Table 1. Considering the effects of ecological variables on the mosquito life cycle [16, 17], we selected predictors to include in the models. To evaluate the robustness of the nowcasting, we developed two separate sets of machine learning models using distinct predictor sets, “Variable Set 1” and “Variable Set 2.” The “No.” column in Table 1 indicates the identifier assigned to each predictor variable for ease of reference.

### 2.4. Machine Learning Models

We developed predictive models using machine learning methodologies, including gradient boosting models (GBMs) and extreme gradient boosting (XGB). Both GBM and XGB are ensemble learning techniques that combine decision tree models with the boosting method [18]. These models iteratively learn from the data to reduce residuals, following a boosting approach. GBM operates by sequentially linking multiple decision trees and adding new trees at each training stage to compensate for the errors of the previous trees, resulting in a powerful model with high predictive accuracy and the ability to effectively capture complex patterns. XGB is a modification of GBM that constructs faster and more accurate models through parallel processing and efficient algorithm implementations. Studies predicting disease occurrence risk by leveraging various predictors with spatial attributes have shown that these models exhibit reliable performance [19]. Additionally, we fitted an ensemble model combining GBM and XGB to enhance predictive performance. The models nowcasted the log-transformed mosquito abundance for each collection site using meteorological factors combined with the log-transformed mosquito abundances from previous weeks (Table 1). The descriptive statistics of the predictors used in the models are presented in Table S2. We used data from 2009 to 2021 as the training dataset and data from 2022 as the test dataset to prevent overfitting and objectively evaluate model performance. This temporal split is a standard practice in time-series modeling [9], ensuring that the model is trained on past data and tested on independent, future data. Since the goal of this study is to develop a mosquito abundance prediction model for practical application, using a temporally ordered test dataset allows us to validate the model's ability to predict mosquito abundance under real-world conditions where future trends are inherently unknown. Table S3 presents the hyperparameters for GBM and XGB. In cases where mosquito abundance was 0, we replaced the value with 0.1 to prevent issues during log transformation. To address the random variability of mosquito abundance, we utilized a moving average (current, 1-week ago, or 2-weeks ago).

We validated model performance using root mean square error (RMSE) and *R*-squared value (R^2^) [20], calculated by comparing actual observational values with model-predicted values. We employed these metrics to provide a robust evaluation and prevent underestimation of model performance due to frequent 0 values in mosquito count data.

### 2.5. Predicting the Peak of Malaria Cases Based on Mosquito Abundance Data

We defined the epidemic peak of malaria based on *R*_t_ and examined whether mosquito abundance data could effectively predict it. *R*_t_ measures the transmission potential and is defined as the average number of secondary cases produced by a single infected person within a population [12]. It is commonly used to assess surveillance systems and intervention programs for vector-borne diseases, including malaria [21]. We calculated *R*_t_ based on the aggregated weekly malaria cases at the provincial level using the backward-looking method [22]. The formula is as follows:

  $$\begin{array}{c} R_{t}\;=\frac{I_{t}}{\sum_{s\text{⁣}=\text{⁣}1}^{t}I_{t-s}W\left(s\right)}, \end{array}$$

  $$\begin{array}{c} W\left(s\right)\;=\left[\frac{1}{\gamma \left(a\right)\theta^{a}}\right]s^{a-1}e^{-\frac{s}{\theta }}, \end{array}$$

where *R*_*t*_ is the reproduction number, *t* is the number of days elapsed since the start of the epidemic, *I*_t_ is the number of cases on day *t*, *W* (*s*) is the current infectivity on day *s* after infection, *a* is the shape parameter, and *θ* is the scale parameter. To estimate current infectivity *W* (*s*) while calculating *R*_t_, we utilized the serial interval and standard deviation for *P. vivax* malaria [23]. The serial interval is a key epidemiological metric that reflects the infectivity and transmission dynamics of an infectious disease. It represents the average time between the onset of symptoms in an initial case and the appearance of symptoms in a secondary case.

**Figure 3:**
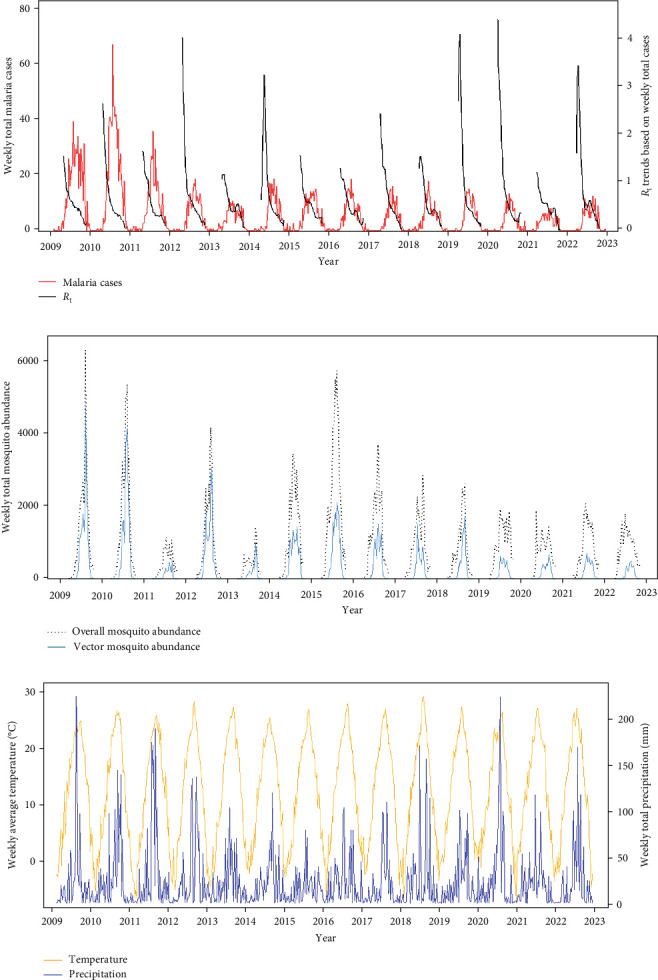
Time-series plots illustrating relationships among key variables. (A) Weekly total malaria cases reported across the entire study area between 2009 and 2022. The *R*_t_ was calculated based on these cases. (B) Dynamic changes in overall number of mosquitoes and malaria vector mosquito abundance collected during the study period. (C) Trends of weekly average ambient temperature and weekly total precipitation over the study period.

Then, we fitted a generalized linear model (GLM) for each province, with log-transformed weekly *R*_t_ as the response variable and log-transformed weekly vector mosquito abundances as the explanatory variable. For each year, we conducted the analysis using data from the periods when *R*_t_ was ≥1 (Figures S3, S4). Considering that the epidemic curve typically has one peak per year (Figure 3A), the moment when *R*_t_ falls below 1 can be considered the epidemic peak of that year. Thus, if the GLMs demonstrate a reliable fit, mosquito abundance may be able to predict when malaria cases will peak. The following equation is the mathematical expression of the model:

  
$$\begin{array}{c} \log\left(R_{t}\right)=\alpha_{0}+\beta_{1}\log\left(M_{t-n}\right)+\beta_{2}\text{week}+\beta_{3}\text{year}+\varepsilon , \end{array}$$

where *R*_*t*_ represents the effective reproduction number, *α*_0_ is the constant intercept, *β*_*n*_ is the regression coefficient, *M*_t-n_ denotes mosquito abundance from previous weeks, “week” refers to the week number to adjust for seasonality, and “year” represents the year to distinguish between years. The model's explanatory power was evaluated using the significance of the regression coefficient (*β*_1_) and *R*^2^. The methods related to the calculation and application of *R*_t_ were detailed in a previous paper [24].

## 3. Results

Figure 2 presents the distribution of the cumulative number of malaria cases and mosquitoes collected between 2009 and 2022. During this period, 4392 malaria cases were reported in the study area, with the highest incidence per 100,000 population occurring in northern Gyeonggi-do. In total, 444,956 mosquitoes were collected, of which 157,219 were malaria vector mosquitoes. Notably, areas with high mosquito abundance differed from the hotspots for human malaria cases. While the pattern of malaria occurrence remained consistent throughout the study period, the distribution of mosquito abundances varied annually (Figures S5–S7).

Malaria cases and mosquito abundance showed clear seasonality, with an increase around April and nearly zero cases after November (Figures 3A,B and S8). *R*_t_ consistently peaked prior to the peak of malaria cases each year, suggesting that it could serve as an indicator for malaria epidemic peaks (Figure 3A). Additionally, the increase in malaria cases often precedes the increase in vector mosquito abundance (Figure S8). Therefore, instead of predicting the onset of malaria cases, it appears more effective to forecast the peak period of malaria as defined by *R*_t_. Fluctuations in ambient temperature and precipitation appear to be correlated with the seasonality of malaria cases and mosquito abundance (Figure 3C). The time series data illustrating associations among the variables are described in detail in Figures S9–S15.

**Figure 4:**
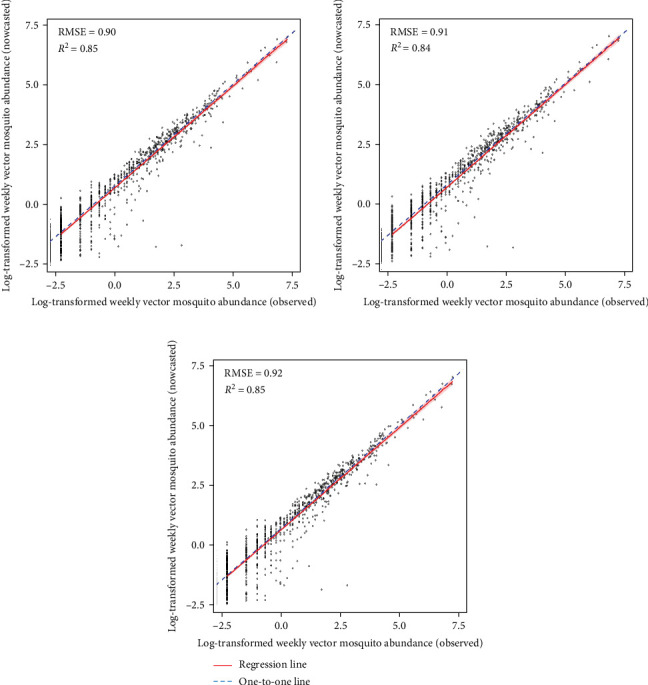
Predictive performance of machine learning models nowcasting vector mosquito abundance. Scatter plot of the association between observed and nowcasted values of vector mosquito abundance. The red line visualizes the linear regression of the scatter plot; the one-to-one line, where observed values match nowcasted values, is indicated by a blue dotted line. (A–C) Performances of the ensemble, GBM, and XGB models, respectively.

Machine learning models reliably nowcasted log-transformed vector mosquito abundance using meteorological factors combined with the log-transformed mosquito abundances from 2 weeks prior (Figure 4). The ensemble models showed the best performance in nowcasting mosquito abundance, with an RMSE of 0.90 and an *R*^2^ value of 0.85. The RMSE and *R*^2^ were 0.91 and 0.84 for the GBM model and 0.92 and 0.85 for the XGB model, respectively. No significant differences in predictive performance were evident between variable sets one and two (Tables S4–S6). The greatest predictive accuracy was observed when the moving average of mosquito abundance over the previous 3 weeks served as an input. A model is generally assumed to adequately explain the majority of the data when it has an R^2^ > 0.7 [24]. Additionally, the range of vector mosquito abundance data extends from 0 to 2,398, with an average value of 23.68 (on the same scale as RMSE). Therefore, the models appear reliable for nowcasting vector mosquito abundance. The results of visualizing the predicted and observed values for each model are depicted in Figures S16–S18.

**Figure 5:**
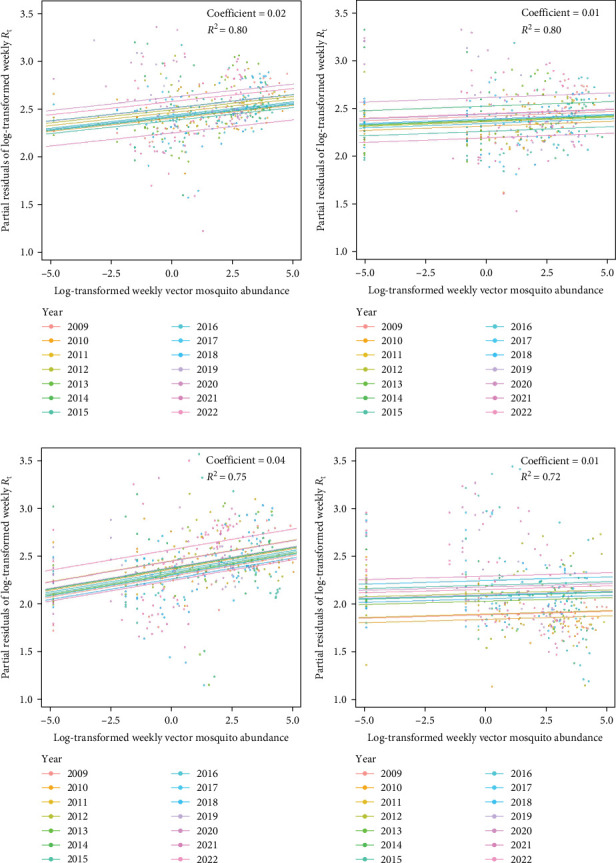
Performances of GLMs examining the effects of vector mosquito abundance on *R*_t_. Scatter plots and regression lines represent the association between log-transformed vector mosquito abundance 2 weeks prior and partial residuals of log-transformed *R*_t_ for each year. Data were collected during periods in which *R*_t_ was ≥1 per year. (A) Aggregated data for the entire study area. (B–D) Results for Gyeonggi-do, Incheon-si, and Gangwon-do, respectively.

The predictive performance of GLMs was generally fair, and vector mosquito abundance appears to be a significant indicator for malaria epidemic peaks. The models forecasting *R*_t_ 2 weeks in advance using mosquito abundance showed a significant association between log-transformed *R*_t_ and log-transformed mosquito abundance (Figure 5 and Table S7). To examine the associations by year, while adjusting for the effect of the week (seasonality), we used partial residuals of log-transformed *R*_t_. In all regions, the coefficient for the mosquito abundance variable was statistically significant and exhibited an *R*_t_ > 0.7, which is generally considered fair. Gangwon-do showed the lowest predictive performance, likely due to the presence of fewer malaria cases compared with other regions. The results of fitting GLMs for each region, using various combinations of forecast periods and lengths of moving averages, are presented in Tables S8–S11.

## 4. Discussion

Using mosquito surveillance data and human malaria cases collected between 2009 and 2022 in the ROK, our study provides valuable insights for developing malaria management policies in malaria-endemic regions. First, we proposed a reliable model for nowcasting mosquito abundance, driven by various meteorological factors. Considering the reporting lag that prevents access to real-time mosquito data, the estimated mosquito abundance from our model offers crucial components for establishing timely vector control strategies. Second, we suggested that mosquito abundance could be an indicator of malaria epidemic peaks. Thus, our findings are expected to contribute to establishing an effective malaria control system by accessing real-time vector surveillance data and utilizing it to identify regions and periods at high risk for malaria outbreaks.

Vector mosquito abundance has been identified as an important determinant of malaria epidemic peaks based on the results of GLMs, which use *R*_t_ as the response variable and vector mosquito abundance from previous weeks as the main explanatory variable. Furthermore, a higher number of malaria cases may create more opportunities for disease transmission, sustaining an elevated *R*_t_ and accelerating the spread of the epidemic [12]. However, the size and timing of the epidemic peak cannot be determined solely by the number of malaria cases. Vector mosquito abundance, as a key driver of malaria transmission, also plays a critical role in shaping both the timing and magnitude of the epidemic. Notably, the association between vector mosquito abundance and *R*_t_ may vary depending on the number of cases. For instance, when the number of cases is extremely low or extremely high, the association can differ from patterns observed within a moderate range of cases. When the number of infected individuals is small, high mosquito abundance can sustain or even steeply increase *R*_t_, facilitating local transmission and potentially leading to a gradual rise in cases. In this scenario, mosquito abundance strongly influences the spread of malaria, amplifying transmission from the limited existing cases. Conversely, when the number of infected individuals is very high, the role of mosquito abundance becomes more context dependent. If mosquito abundance is sufficient to sustain transmission, *R*_t_ may remain above 1, driving a rapid increase in cases until the epidemic peak is reached. However, if mosquito abundance is insufficient relative to the number of infected individuals, transmission efficiency may decline due to increased competition among mosquitoes for infected hosts. These dynamics contribute to the eventual peak and subsequent decline of the epidemic.

Our model could forecast the timing and location of surges in malaria vector mosquitoes. The results can be used to enhance the timing of insecticide fogging distribution for controlling adult mosquitoes. These conventional interventions remain effective, particularly during epidemic seasons [25]. Intensive insecticide application in residential areas or livestock farms can lead to short-term reductions in mosquito abundance, thereby, preventing potential malaria outbreaks [26]. The existing vector control programs assume a consistent growth curve in vector mosquito abundance and malaria cases over the years [26]. However, our descriptive analysis (Figures S3, S4), and a previous study [27] revealed that the peak times of mosquito abundance and malaria cases were not consistent across years. In this regard, our models can be utilized in a practical manner by health authorities to design optimal control measures for reducing mosquito abundance.

Reduction of mosquito surveillance reporting lag is essential for efforts to expand our model into a larval control program. Control strategies that combine larval source management are more effective than those primarily targeting adult mosquitoes [28]. This effectiveness arises because mosquitoes cluster in water-containing habitats during their larval and pupal stages [29], making it easier to identify and target these habitats for control measures. However, constraints exist when applying our results to conduct control programs focused on the larval stage. Considering that the development period from larva to adult mosquitoes is approximately 1–2 weeks [29], at least a 2-week forecast of adult mosquito abundance is required to optimize the timing of larval control. Although the model can predict mosquito abundance 2 weeks in advance, the mosquito surveillance data have a reporting delay of 2 weeks. Thus, the most recent mosquito abundance data are those collected 2 weeks prior. By utilizing cutting-edge technologies such as smart mosquito traps [30] for real-time mosquito abundance monitoring, our model can be used to forecast adult mosquito abundance 2 weeks later. This would allow for the implementation of control measures at the appropriate time to prevent the larval stage from metamorphosing into adult mosquitoes.

We demonstrated that mosquito abundance can serve as a predictor of malaria epidemic peaks. In our statistical model, the *R*_t_ of malaria in the human population was effectively predicted by mosquito abundance from 2 weeks earlier. This finding suggests that mosquito abundance plays a crucial role in the secondary attack rates of human infections transmitted by infected mosquitoes. Considering that determination of the optimal timing for malaria control measure implementation is crucial for the efficacy [31] and cost-effectiveness [32] of these measures, our findings could be integrated into an early warning system to improve malaria control strategies by offering reliable information concerning mosquito abundance and its influence on malaria epidemics. Despite the high *R*^2^ values of our models (generally around 0.8), more advanced modeling is required to use the models for practical forecasting of malaria epidemics based on mosquito abundance. For improved predictive capabilities, we strongly recommend the inclusion of additional predictors such as land use [33], temperature, humidity, precipitation [9], and extreme weather events [34]. Through such processes, the enhanced model could contribute to building malaria early warning systems in malaria-endemic regions [35].

Several limitations of this study warrant consideration. First, our nowcasting models for mosquito abundance incorporated the 3-week moving average of mosquito abundance as a predictive factor. We employed this moving average to mitigate potential errors in the mosquito counting process. However, this approach may have overestimated the performances of our nowcasting models because we used mosquito abundance data from 2 weeks prior as a predictor, which coincides with the period covered by the moving average. Second, the history of mosquito control implementation was not considered in our nowcasting of mosquito abundance. Incorporation of parameters that reflect these control measures would enhance the model's predictive capacity. Third, our descriptive analysis revealed considerable variation in mosquito abundance across collection sites. Certain sites displayed excess zero values, potentially facilitating easier prediction of abundances at these locations compared with other locations. Consequently, this may lead to an overestimation of the models' performances. Finally, it is crucial to acknowledge that the efficiency of mosquito collection differs between black light traps and LED traps. LED traps proved more efficient in collecting a larger and more varied mosquito population compared with black light traps [36], potentially introducing systematic bias into our analysis. Therefore, to prevent incorrect conclusions drawn from trapping methods, further assessments that consider host attraction and trapping techniques are necessary.

## 5. Conclusion

We have introduced a reliable model for nowcasting mosquito abundance, which could be incorporated into a malaria early warning system. Our models, which nowcast mosquito abundance based on various meteorological factors, provide evidence for the effective application of vector control measures. Furthermore, we suggest that mosquito abundance could predict malaria epidemic peaks. These results offer valuable insights for the development of malaria management policies in the ROK, where ongoing control activities have maintained *P. vivax* malaria at a steady-state level, but progress toward elimination has been slow. Therefore, our findings may be implemented in regions where malaria eradication presents a significant and immediate challenge.

## Figures and Tables

**Table 1:** Predictors included in machine learning models.

No.	Predictor set 1	Predictor set 2
1	The difference between the daily average temperature and the optimal temperature for mosquito growth (27.5°C) in the current week.	The difference between the daily average temperature and the optimal temperature for mosquito growth (27.5°C) in the current week.
2	The difference between the daily average temperature and the optimal temperature for mosquito growth (27.5°C) over the previous 2 weeks.	—
3	Weekly mean ambient temperature in the current week.	Weekly mean ambient temperature in the current week.
4	Weekly total precipitation in the current week.	Weekly total precipitation in the current week.
5	Weekly mean sunshine duration in the current week.	Weekly mean sunshine duration in the current week.
6	Weekly mean relative humidity in the current week.	Weekly mean relative humidity in the current week.
7	Weekly mean ambient temperature in the previous 2 weeks.	—
8	Weekly total precipitation in the previous 2 weeks.	—
9	Weekly mean sunshine duration in the previous 2 weeks.	—
10	Weekly mean relative humidity in the previous 2 weeks.	—
11	The number of days with heavy precipitation (97th percentile of precipitation distribution) in the current week.	—
12	The number of days with light precipitation (20 mm or less) in the current week.	The number of days with light precipitation (20 mm or less) from the previous week.
13	The number of days with heavy precipitation (97th percentile of precipitation distribution) over the previous 2 weeks.	—
14	The number of days with light precipitation (20 mm or less) over the previous 2 weeks.	—
15	Mosquito abundance data from 2 weeks ago.	Mosquito abundance data from 2 weeks ago.
16	Mosquito abundance data from 3 weeks ago.	Mosquito abundance data from 3 weeks ago.
17	Mosquito abundance data from the same week 1 year ago.	Mosquito abundance data from the same week 1 year ago.
18	Mosquito abundance data from 1 week before the same week 1 year ago.	Mosquito abundance data from 1 week before the same week 1 year ago.
19	Mosquito abundance data from 2 weeks before the same week 1 year ago.	Mosquito abundance data from 2 weeks before the same week 1 year ago.

## Data Availability

The authors have ensured the reproducibility of the analysis by providing the sources of the public data used in the study and including the underlying information in the Supporting Information. Additionally, data supporting this study's findings are available from the corresponding author upon reasonable request.

## Nomenclature

DMZ: demilitarized zone
GBM: gradient boosting model
GLM: generalized linear model
KDCA: Korea Disease Control and Prevention Agency
LED: light-emitting diode
*R*_t_: effective reproduction number
*R* ^2^: *R*-squared value
RMSE: root mean square error
ROK: Republic of Korea
XGB: extreme gradient boosting.

## Supporting Information

Supporting Information Table S1. Changes in the number of mosquito collection sites. Table S2. Descriptive statistics of the predictors included in predictor sets. The numbers in the ‘No.' column correspond to the numbers in the ‘No.' column of Table 1. Table S3. Hyperparameters used in the machine learning models in this study. Table S4. The predictive power of the ensemble models for nowcasting log-transformed mosquito abundances based on data from 2 weeks prior. Table S5. The predictive power of the GBMs for nowcasting log-transformed mosquito abundances based on data from 2 weeks prior. Table S6. The predictive power of the XGBs for nowcasting log-transformed mosquito abundances based on data from 2 weeks prior. Table S7. Predictive power of GLMs that nowcasting the log effective reproduction number (RRt) based on vector mosquito abundances. Table S8. The predictive power of GLMs that forecast the log effective reproduction number (RRt) based on vector mosquito abundances, with aggregated data for the entire region. Table S9. The predictive power of GLMs that forecast the log effective reproduction number (RRt) based on vector mosquito abundances, with data for Gyeonggi-do. Table S10. The predictive power of GLMs that forecast the log effective reproduction number (RRt) based on vector mosquito abundances, with data for Incheon-si. Table S11. The predictive power of GLMs that forecast the log effective reproduction number (RRt) based on vector mosquito abundances, with data for Gangwon-do. Figure S1. Map of the nationwide malaria cases. Figure S2. Map of the nationwide meteorological stations. Figure S3. The timing of peak malaria cases annually 1. Figure S4. The timing of peak malaria cases annually 2. Figure S5. The distribution of annual malaria cases in the study area from 2009 to 2022. Figure S6. The distribution of annual abundance of overall mosquitos in the study area from 2009 to 2022. Figure S7. The distribution of annual abundance of vector mosquitos in the study area from 2009 to 2022. Figure S8. Monthly aggregated malaria cases and vector mosquito abundances from 2009 to 2022. Figure S9. Weekly total malaria cases from 2009 to 2022. Figure S10. Calculated RRt based on weekly total malaria cases from 2009 to 2022. Figure S11. Weekly total malaria cases and RRt based on weekly total malaria cases from 2009 to 2022. Figure S12. Weekly total overall and vector mosquito abundance from 2009 to 2022. Figure S13. Weekly total malaria cases and vector mosquito abundance from 2009 to 2022. Figure S14. Calculated RRt based on weekly total malaria cases and weekly total vector mosquito abundance from 2009 to 2022. Figure S15. Weekly average ambient temperature and weekly total precipitation from 2009 to 2022. Figure S16. Visualization of predicted and observed values of vector mosquito abundance based on GBMs. Figure S17. Visualization of predicted and observed values of vector mosquito abundance based on XGBs. Figure S18. Visualization of predicted and observed values of vector mosquito abundance based on ensemble models.
