# Supplementary material for: Nowcasting Vector Mosquito Abundance and Determining Its Association With Malaria Epidemics in South Korea
Source: Transbound Emerg Dis. 2025 Jan 17;2025:9959287. doi: 10.1155/tbed/9959287 (PMC12016956; doi:10.1155/tbed/9959287)
Supplement: Supporting Information — Table S1. Changes in the number of mosquito collection sites. Table S2. Descriptive statistics of the predictors included in predictor sets. The numbers in the ‘No.' column correspond to the numbers in the ‘No.' column of Table 1. Table S3. Hyperparameters used in the machine learning models in this study. Table S4. The predictive power of the ensemble models for nowcasting log-transformed mosquito abundances based on data from 2 weeks prior. Table S5. The predictive power of the GBMs for nowcasting log-transformed mosquito abundances based on data from 2 weeks prior. Table S6. The predictive power of the XGBs for nowcasting log-transformed mosquito abundances based on data from 2 weeks prior. Table S7. Predictive power of GLMs that nowcasting the log effective reproduction number (RRt) based on vector mosquito abundances. Table S8. The predictive power of GLMs that forecast the log effective reproduction number (RRt) based on vector mosquito abundances, with aggregated data for the entire region. Table S9. The predictive power of GLMs that forecast the log effective reproduction number (RRt) based on vector mosquito abundances, with data for Gyeonggi-do. Table S10. The predictive power of GLMs that forecast the log effective reproduction number (RRt) based on vector mosquito abundances, with data for Incheon-si. Table S11. The predictive power of GLMs that forecast the log effective reproduction number (RRt) based on vector mosquito abundances, with data for Gangwon-do. Figure S1. Map of the nationwide malaria cases. Figure S2. Map of the nationwide meteorological stations. Figure S3. The timing of peak malaria cases annually 1. Figure S4. The timing of peak malaria cases annually 2. Figure S5. The distribution of annual malaria cases in the study area from 2009 to 2022. Figure S6. The distribution of annual abundance of overall mosquitos in the study area from 2009 to 2022. Figure S7. The distribution of annual abundance of vector mosquitos in the study area from [file 9959287.f1.docx]

**Supporting Information**

**Page 2 – Table S1.** Changes in the number of mosquito collection sites.

**Page 3 – Table S2.** Descriptive statistics of the predictors included in Predictor Sets. The numbers in the 'No.' column correspond to the numbers in the 'No.' column of Table 1.

**Page 5 – Table S3.** Hyperparameters used in the machine learning models in this study.

**Page 6 – Table S4.** The Predictive power of the ensemble models for nowcasting log-transformed mosquito abundances based on data from two weeks prior.

**Page 7 – Table S5.** The predictive power of the GBMs for nowcasting log-transformed mosquito abundances based on data from two weeks prior.

**Page 8 – Table S6.** The predictive power of the XGBs for nowcasting log-transformed mosquito abundances based on data from two weeks prior.

**Page 9 – Table S7.** Predictive power of GLMs that nowcasting the log effective reproduction number (R_t_) based on vector mosquito abundances.

**Page 10 – Table S8.** The predictive power of GLMs that forecast the log effective reproduction number (R_t_) based on vector mosquito abundances, with aggregated data for the entire region.

**Page 11 – Table S9.** The predictive power of GLMs that forecast the log effective reproduction number (R_t_) based on vector mosquito abundances, with data for Gyeonggi-do.

**Page 12 – Table S10.** The predictive power of GLMs that forecast the log effective reproduction number (R_t_) based on vector mosquito abundances, with data for Incheon-si.

**Page 13 – Table S11.** The predictive power of GLMs that forecast the log effective reproduction number (R_t_) based on vector mosquito abundances, with data for Gangwon-do.

**Page 14 – Figure S1. Map of the nationwide malaria cases.**

**Page 15 – Figure S2. Map of the nationwide meteorological stations**.

**Page 16 – Figure S3. The timing of peak malaria cases annually 1**.

**Page 17 – Figure S4**. **The timing of peak malaria cases annually 2**.

**Page 18 – Figure S5. The distribution of annual malaria cases in the study area from 2009 to 2022.**

**Page 19 – Figure S6. The distribution of annual abundance of overall mosquitos in the study area from 2009 to 2022**.

**Page 20 – Figure S7. The distribution of annual abundance of vector mosquitos in the study area from 2009 to 2022**.

**Page 21 – Figure S8**. **Monthly aggregated malaria cases and vector mosquito abundances from 2009 to 2022**.

**Page 22 – Figure S9. Weekly total malaria cases from 2009 to 2022.**

**Page 23 – Figure S10. Calculated R_t_ based on weekly total malaria cases from 2009 to 2022**.

**Page 24 – Figure S11. Weekly total malaria cases and R_t_ based on weekly total malaria cases from 2009 to 2022**.

**Page 25 – Figure S12**. **Weekly total overall and vector mosquito abundance from 2009 to 2022**.

**Page 26 – Figure S13. Weekly total malaria cases and vector mosquito abundance from 2009 to 2022.**

**Page 27 – Figure S14. Calculated R_t_ based on weekly total malaria cases and weekly total vector mosquito abundance from 2009 to 2022**.

**Page 28 – Figure S15. Weekly average ambient temperature and weekly total precipitation from 2009 to 2022**.

**Page 29 – Figure S16**. **Visualization of predicted and observed values of vector mosquito abundance based on GBMs**.

**Page 30 – Figure S17. Visualization of predicted and observed values of vector mosquito abundance based on XGBs.**

**Page 31 – Figure S18. Visualization of predicted and observed values of vector mosquito abundance based on ensemble models.**

**Table S1.** Changes in the number of mosquito collection sites.

| Years | Civilian collection sites | Military collection sites | Total |
| --- | --- | --- | --- |
| 2009 - 2018 | 20 | 0 | 20 |
| 2019 | 32 | 12 | 44 |
| 2020 | 36 | 15 | 51 |
| 2021 - 2023 | 36 | 14 | 50 |

**Table S2.** Descriptive statistics of the predictors included in Predictor Sets. The numbers in the 'No.' column correspond to the numbers in the 'No.' column of Table 1.

| No. | Predictors | Mean | Standard deviation | Range | Median | IQR* |
| --- | --- | --- | --- | --- | --- | --- |
| 1 | The difference between the daily average temperature and the optimal temperature for mosquito growth (27.5 ℃) in the current week. | 7.12 | 4.53 | 0.25 ~ 22.10 | 6.25 | 6.75 |
| 2 | The difference between the daily average temperature and the optimal temperature for mosquito growth (27.5 ℃) over the previous two weeks. | 7.01 | 4.23 | 0.49 ~ 19.01 | 6.19 | 6.73 |
| 3 | Weekly mean ambient temperature in the current week. | 20.48 | 4.67 | 5.40 ~ 31.01 | 21.25 | 6.76 |
| 4 | Weekly mean precipitation in the current week. | 6.08 | 8.92 | 0.00 ~ 101.59 | 2.75 | 7.57 |
| 5 | Weekly mean sunshine duration in the current week. | 6.02 | 5.66 | 1.01 ~ 12.57 | 3.84 | 10.96 |
| 6 | Weekly mean relative humidity in the current week. | 75.62 | 9.66 | 38.72 ~ 98.61 | 76.39 | 13.58 |
| 7 | Weekly mean ambient temperature in the previous two weeks. | 20.59 | 4.38 | 8.49 ~ 30.56 | 21.31 | 6.74 |
| 8 | Weekly mean precipitation in the previous two weeks. | 6.00 | 7.36 | 0.00 ~ 64.66 | 3.45 | 6.38 |
| 9 | Weekly mean sunshine duration in the previous two weeks. | 6.23 | 5.74 | 1.06 ~ 11.46 | 3.94 | 11.69 |
| 10 | Weekly mean relative humidity in the previous two weeks. | 75.20 | 9.30 | 37.64 ~ 97.09 | 75.96 | 13.21 |
| 11 | The number of days with heavy precipitation (97th percentile of precipitation distribution) in the current week. | 0.20 | 0.53 | 0.00 ~ 5.00 | 0.00 | 0.00 |
| 12 | The number of days with light precipitation (20mm or less) in the current week. | 6.28 | 1.09 | 0.00 ~ 7.00 | 6.07 | 1.00 |
| 13 | The number of days with heavy precipitation (97th percentile of precipitation distribution) over the previous two weeks. | 0.22 | 0.58 | 0.00 ~ 5.00 | 0.00 | 0.00 |
| 14 | The number of days with light precipitation (20mm or less) over the previous two weeks. | 12.77 | 1.55 | 5.00 ~ 14.00 | 13.02 | 2.00 |
| 15 | Mosquito abundance data from two weeks ago. | 47.51 | 33.92 | 0.00 ~ 13888.00 | 2.00 | 15.00 |
| 16 | Mosquito abundance data from three weeks ago. | 47.60 | 333.26 | 0.00 ~ 13888.00 | 1.00 | 15.00 |
| 17 | Mosquito abundance data from the same week one year ago. | 54.37 | 353.82 | 0.00 ~ 13888.00 | 2.00 | 17.00 |
| 18 | Mosquito abundance data from one week before the same week one year ago. | 54.40 | 353.85 | 0.00 ~ 13888.00 | 2.00 | 17.00 |
| 19 | Mosquito abundance data from two weeks before the same week one year ago. | 54.34 | 353.81 | 0.00 ~ 13888.00 | 2.00 | 17.00 |

***Interquartile Range**

**Table S3.** Hyperparameters used in the machine learning models in this study.

| Machine-learning models | Hyperparameters |
| --- | --- |
| Gradient boosted models (GBM) | Number of trees = 10,000  learning rate = 0·01  maximum depth of each tree = 4  the fraction of data to be used for each tree's training = 0·9  the minimum number of observations in the terminal nodes of the trees = 10 (default) |
| Extreme gradient boosting (XGB) | Number of trees = 10,000  learning rate = 0·001  maximum depth of each tree = 8  the fraction of data to be used for each tree's training = 0·9  the minimum number of observations in the terminal nodes of the trees = 10 (default) |

**Table S4.** The Predictive power of the ensemble models for nowcasting log-transformed mosquito abundances based on data from two weeks prior.

| No. | Variable set | Moving average | RMSE | R^2^ |
| --- | --- | --- | --- | --- |
| 1 | Set 1 | NA | 1·46 | 0·63 |
| 2 | Set 1 | 2 weeks | 1·21 | 0·73 |
| 3^*^ | Set 1 | 3 weeks | 0·90 | 0·85 |
| 4 | Set 2 | NA | 1·44 | 0·64 |
| 5 | Set 2 | 2 weeks | 1·20 | 0·73 |
| 6 | Set 2 | 3 weeks | 0·89 | 0·85 |

^*^The model is presented in Figure 4.

**Table S5.** The predictive power of the GBMs for nowcasting log-transformed mosquito abundances based on data from two weeks prior.

| No. | Variable set | Moving average | RMSE | R^2^ |
| --- | --- | --- | --- | --- |
| 1 | Set 1 | NA | 1·48 | 0·63 |
| 2 | Set 1 | 2 weeks | 1·24 | 0·73 |
| 3^*^ | Set 1 | 3 weeks | 0·91 | 0·84 |
| 4 | Set 2 | NA | 1·46 | 0·64 |
| 5 | Set 2 | 2 weeks | 1·23 | 0·73 |
| 6 | Set 2 | 3 weeks | 0·91 | 0·85 |

^*^The model is presented in Figure 4.

**Table S6.** The predictive power of the XGBs for nowcasting log-transformed mosquito abundances based on data from two weeks prior.

| No. | Variable set | Moving average | RMSE | R^2^ |
| --- | --- | --- | --- | --- |
| 1 | Set 1 | NA | 1·49 | 0·63 |
| 2 | Set 1 | 2 weeks | 1·22 | 0·73 |
| 3^*^ | Set 1 | 3 weeks | 0·92 | 0·85 |
| 4 | Set 2 | NA | 1·46 | 0·64 |
| 5 | Set 2 | 2 weeks | 1·21 | 0·73 |
| 6 | Set 2 | 3 weeks | 0·92 | 0·85 |

^*^The model is presented in Figure 4.

**Table S7.** Predictive power of GLMs that nowcasting the log effective reproduction number (R_t_) based on vector mosquito abundances.

| No. | Regions | Forecast period | Coefficient of the mosquito abundance (*P*-value) | R^2^ |
| --- | --- | --- | --- | --- |
| 1 | Aggregated for the entire region | 2 weeks | 0·02 (< 0·05) | 0·80 |
| 2 | Gyeonggi-do | 2 weeks | 0·01 (< 0·05) | 0·80 |
| 3 | Incheon-si | 2 weeks | 0·04 (< 0·05) | 0·75 |
| 4 | Gangwon-do | 2 weeks | 0·01 (< 0·05) | 0·72 |

**Table S8.** The predictive power of GLMs that forecast the log effective reproduction number (R_t_) based on vector mosquito abundances, with aggregated data for the entire region.

| No. | Mosquito abundances | Forecast period | Coefficient of the mosquito abundance (*P*-value) | R^2^ |
| --- | --- | --- | --- | --- |
| 1 | Vector mosquito abundance | 1 week | 0·01 (< 0·05) | 0·79 |
| 2 | Vector mosquito abundance | 2 weeks | 0·02 (< 0·05) | 0·80 |
| 3 | Overall mosquito abundance | 1 week | 0·01 (0·15) | 0·80 |
| 4 | Overall mosquito abundance | 2 weeks | 0·04 (0·06) | 0·83 |

**Table S9.** The predictive power of GLMs that forecast the log effective reproduction number (R_t_) based on vector mosquito abundances, with data for Gyeonggi-do.

| No. | Mosquito abundances | Forecast period | Coefficient of the mosquito abundance (*P*-value) | R^2^ |
| --- | --- | --- | --- | --- |
| 1 | Vector mosquito abundance | 1 week | 0·03 (< 0·05) | 0·77 |
| 2 | Vector mosquito abundance | 2 weeks | 0·01 (< 0·05) | 0·80 |
| 3 | Overall mosquito abundance | 1 week | 0·05 (0·16) | 0·80 |
| 4 | Overall mosquito abundance | 2 weeks | 0·03 (0·05) | 0·80 |

**Table S10.** The predictive power of GLMs that forecast the log effective reproduction number (R_t_) based on vector mosquito abundances, with data for Incheon-si.

| No. | Mosquito abundances | Forecast period | Coefficient of the mosquito abundance (*P*-value) | R^2^ |
| --- | --- | --- | --- | --- |
| 1 | Vector mosquito abundance | 1 week | 0·01 (< 0·05) | 0·91 |
| 2 | Vector mosquito abundance | 2 weeks | 0·04 (< 0·05) | 0·75 |
| 3 | Overall mosquito abundance | 1 week | 0·02 (0·10) | 0·70 |
| 4 | Overall mosquito abundance | 2 weeks | 0·03 (0.23) | 0·68 |

**Table S11.** The predictive power of GLMs that forecast the log effective reproduction number (R_t_) based on vector mosquito abundances, with data for Gangwon-do.

| No. | Mosquito abundances | Forecast period | Coefficient of the mosquito abundance (*P*-value) | R^2^ |
| --- | --- | --- | --- | --- |
| 1 | Vector mosquito abundance | 1 week | 0·01 (< 0·05) | 0·75 |
| 2 | Vector mosquito abundance | 2 weeks | 0·01 (< 0·05) | 0·72 |
| 3 | Overall mosquito abundance | 1 week | 0·02 (0·10) | 0·79 |
| 4 | Overall mosquito abundance | 2 weeks | 0·04 (0·23) | 0·70 |


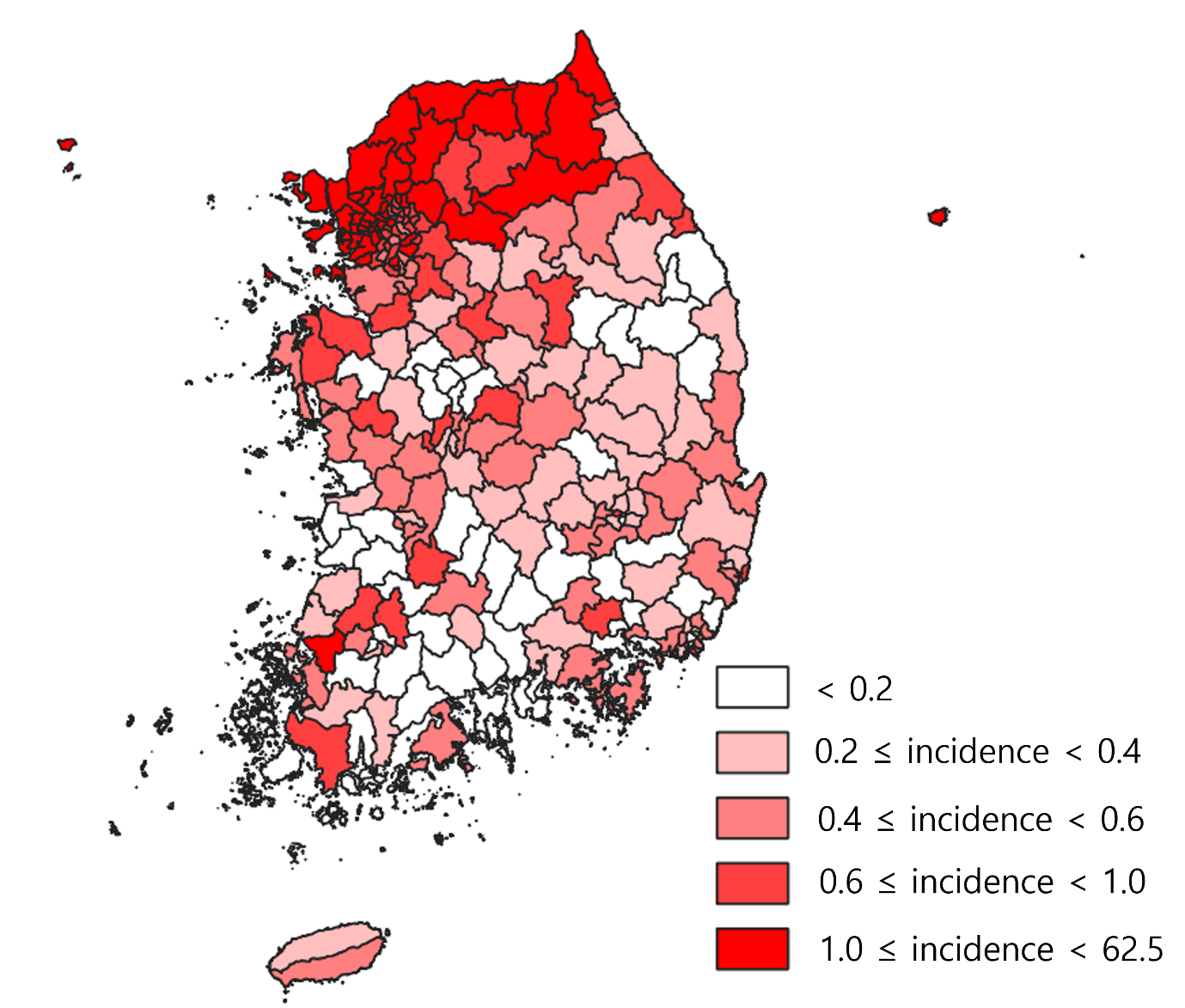


**Figure S1. Map of the nationwide malaria cases.** Average malaria incidence per 100,000 population in the study area between 2009 and 2022.


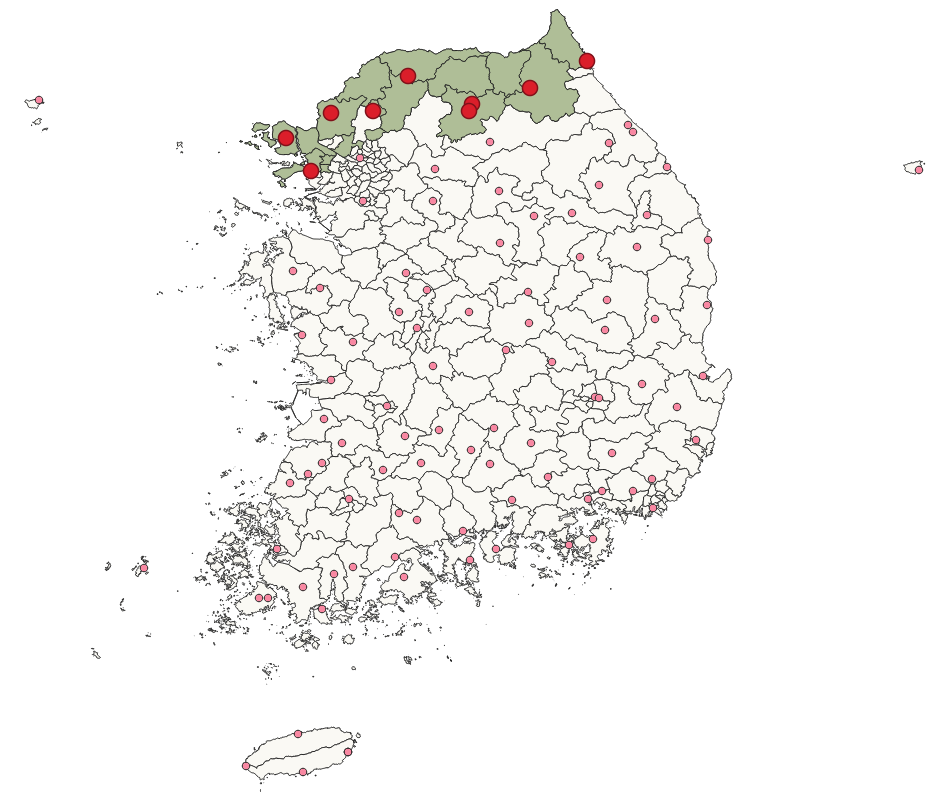


**Figure S2. Map of the nationwide meteorological stations.** 9 red dots indicate the stations in the target regions. There are a total of 103 meteorological stations operating nationwide (red and pink dots).


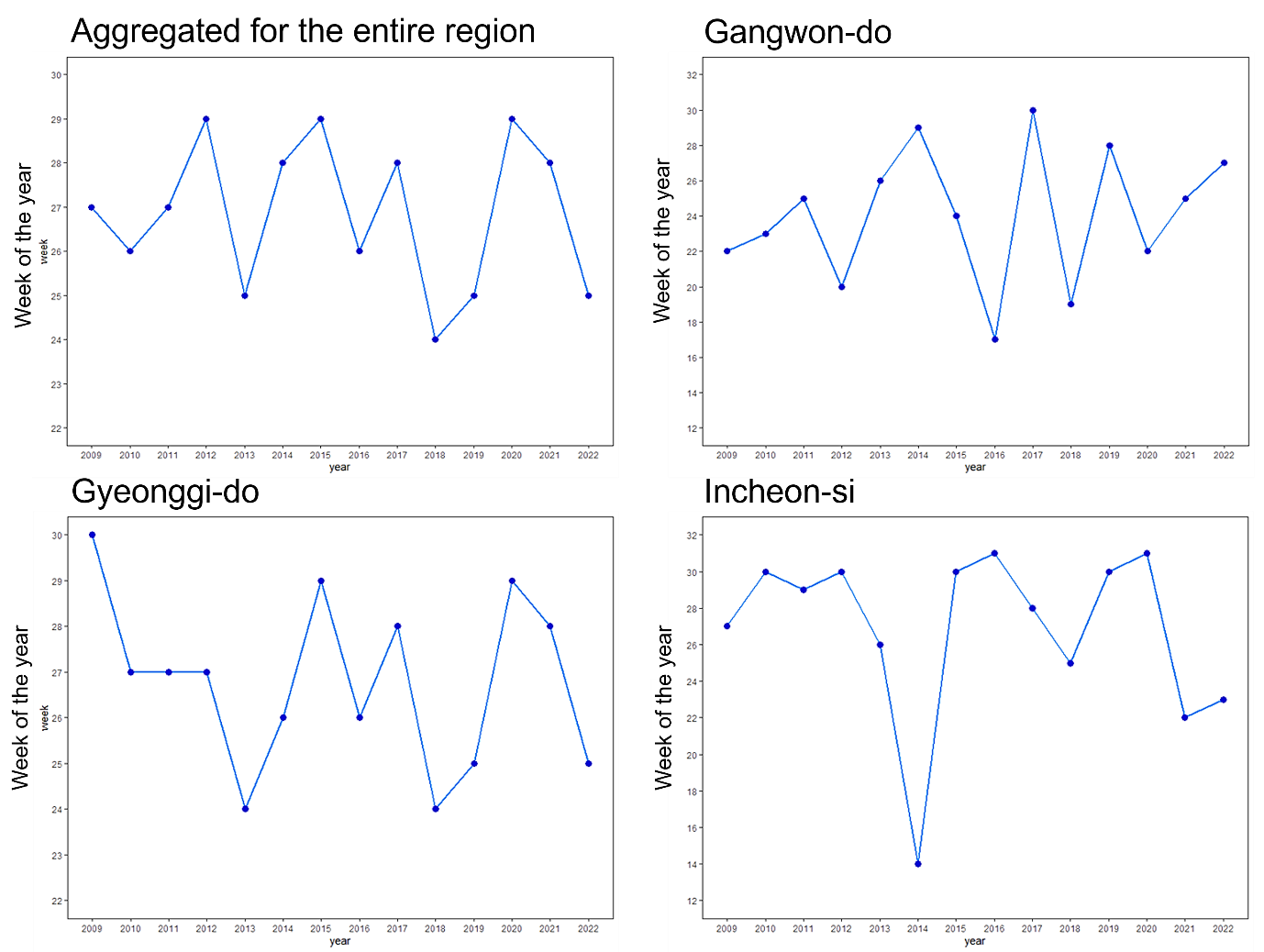


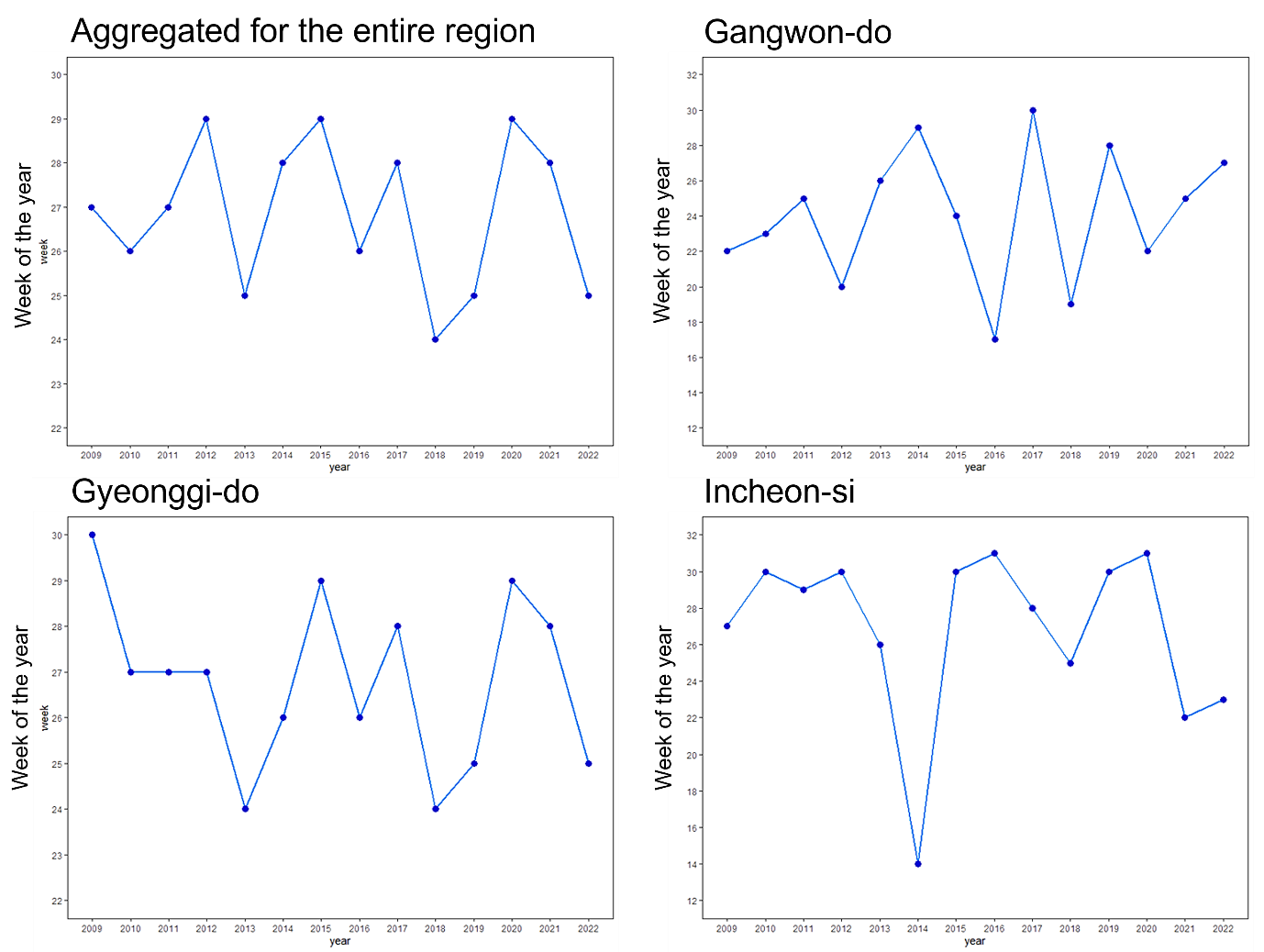


**Figure S3. The timing of peak malaria cases annually.** The weeks in which peak malaria cases occurred from 2009 to 2022 are displayed by region.


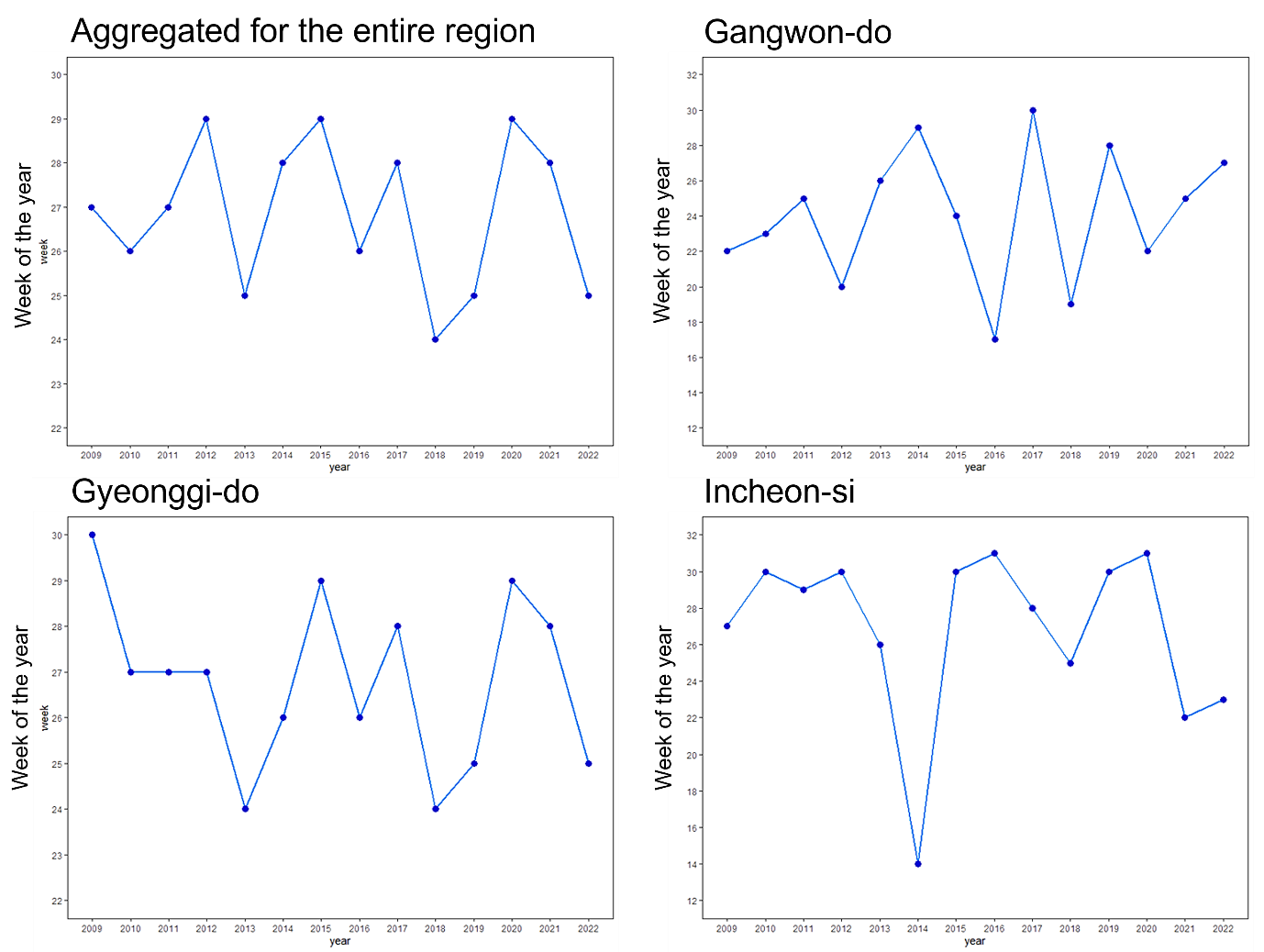

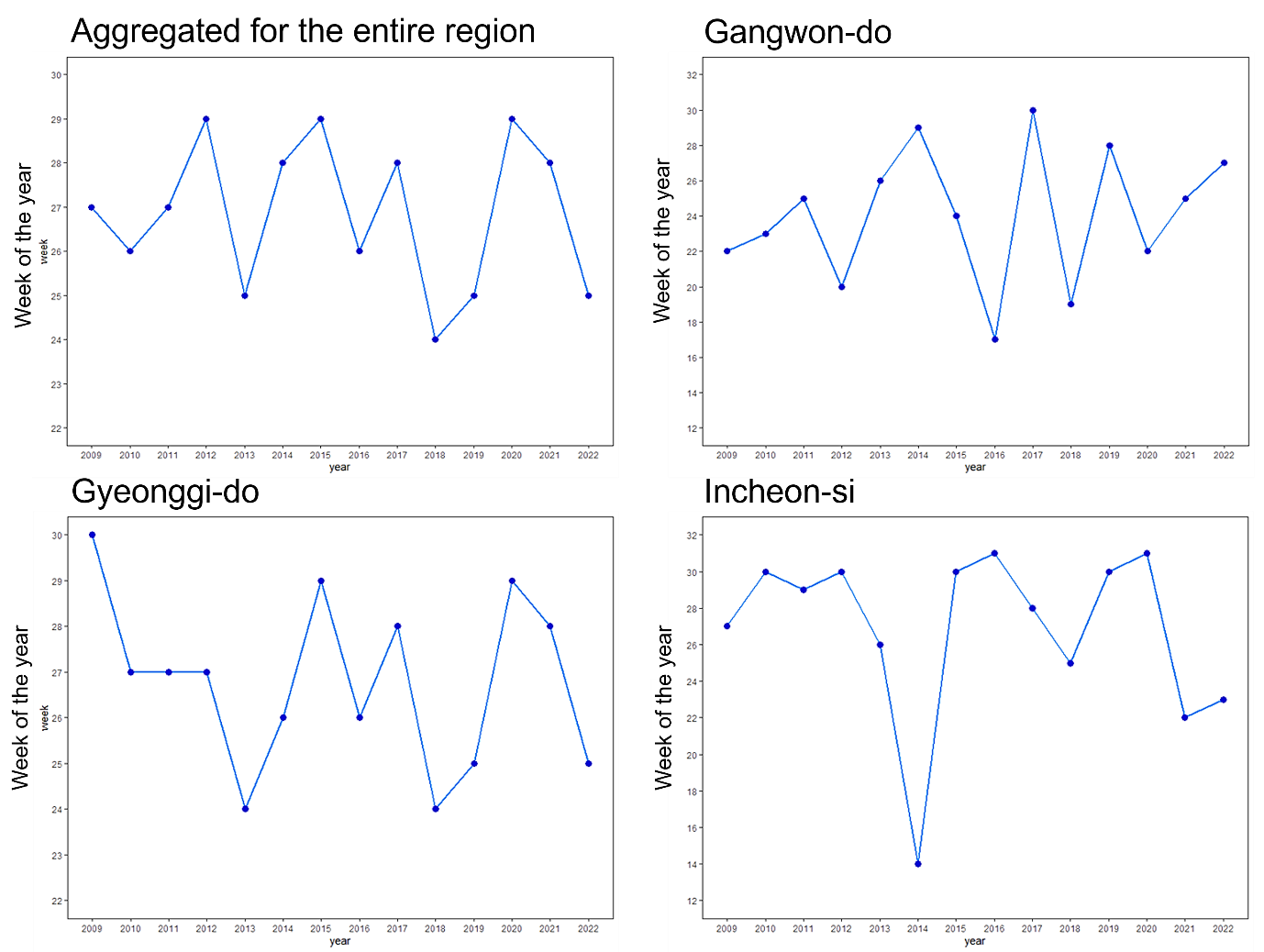


**Figure S4. The timing of peak malaria cases annually.** The weeks in which peak malaria cases occurred from 2009 to 2022 are displayed by region.


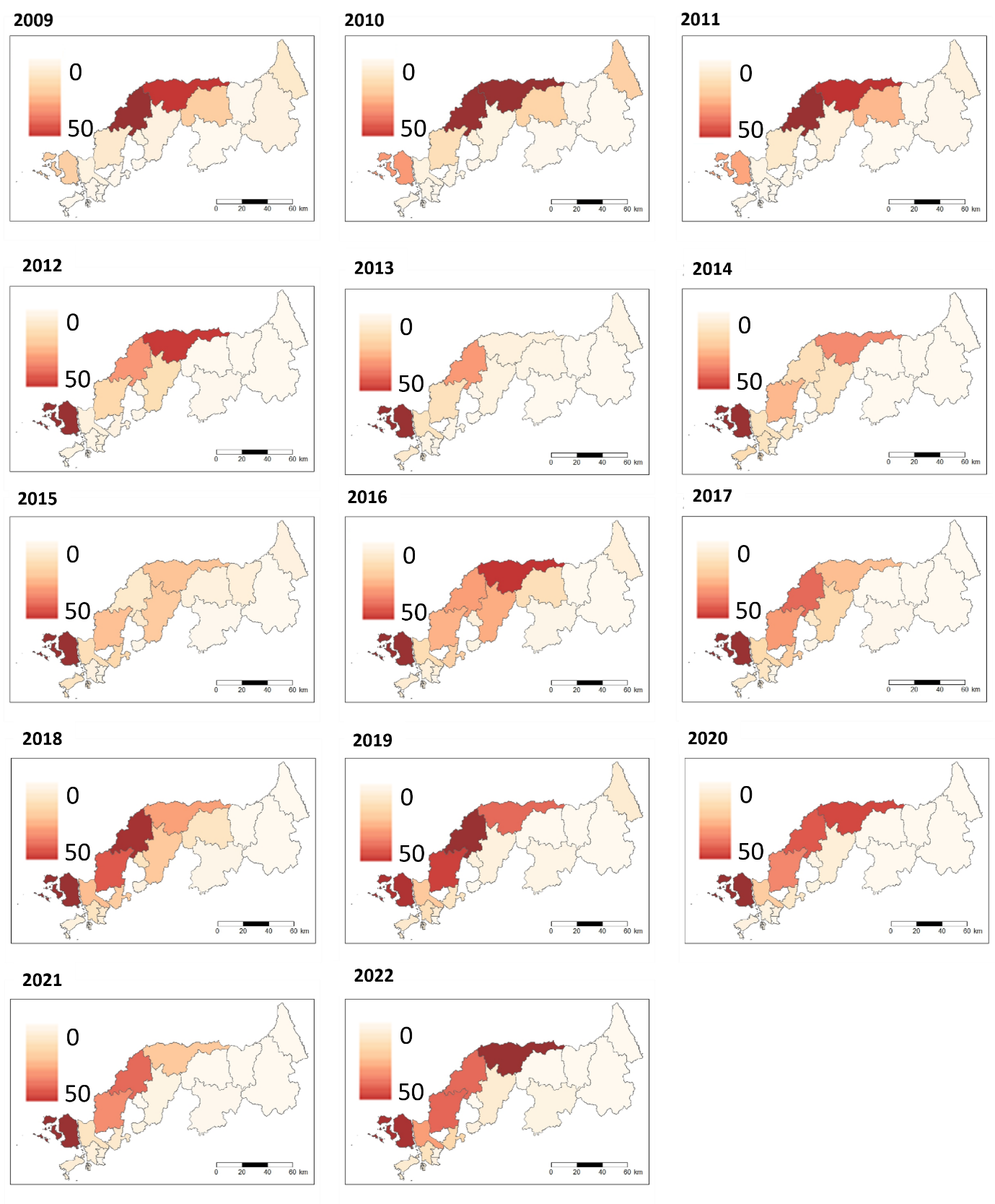


**Figure S5. The distribution of annual malaria incidence per 100,000 population in the study area from 2009 to 2022.**


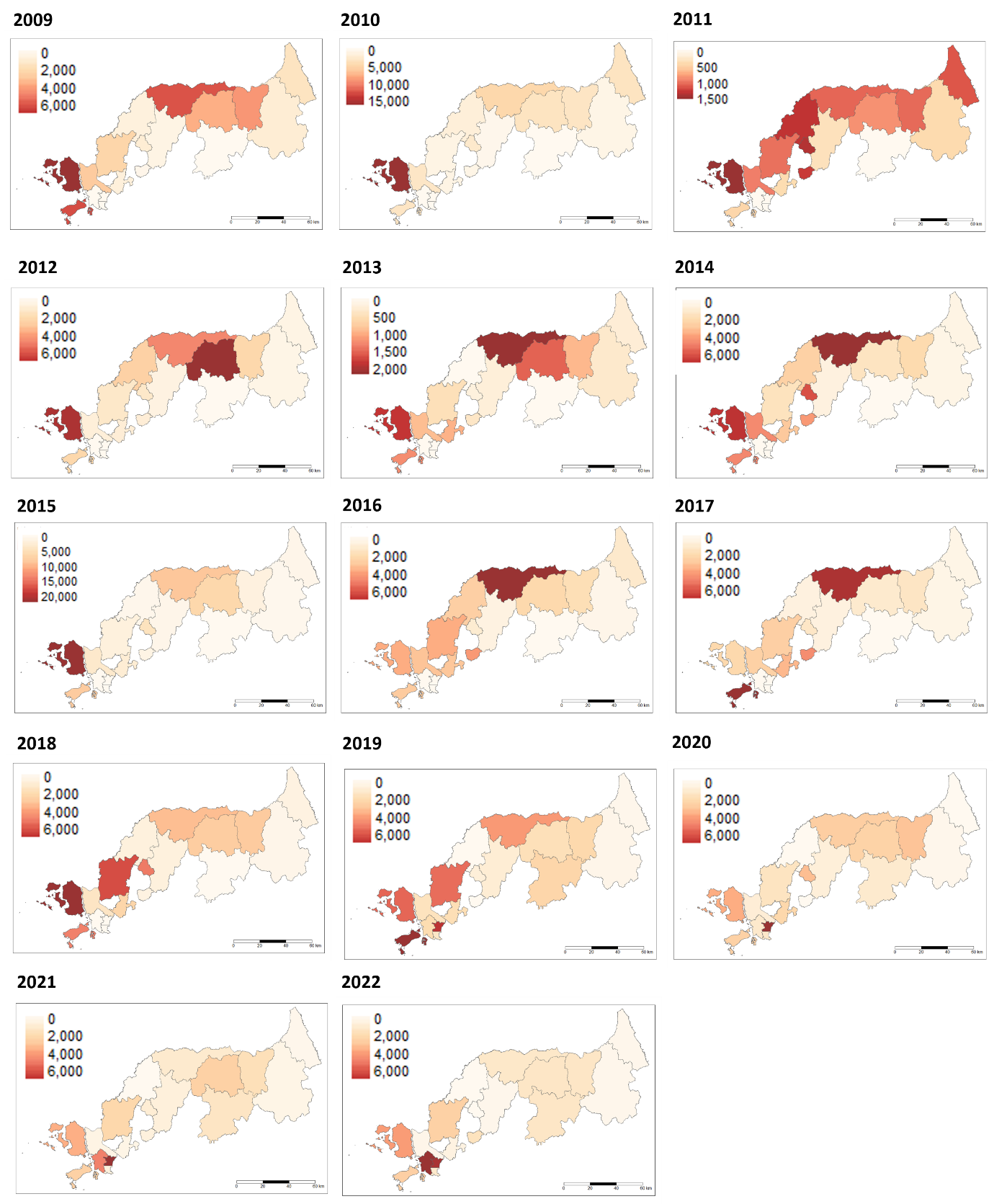


**Figure S6. The distribution of annual abundance of overall mosquitos in the study area from 2009 to 2022.**

**
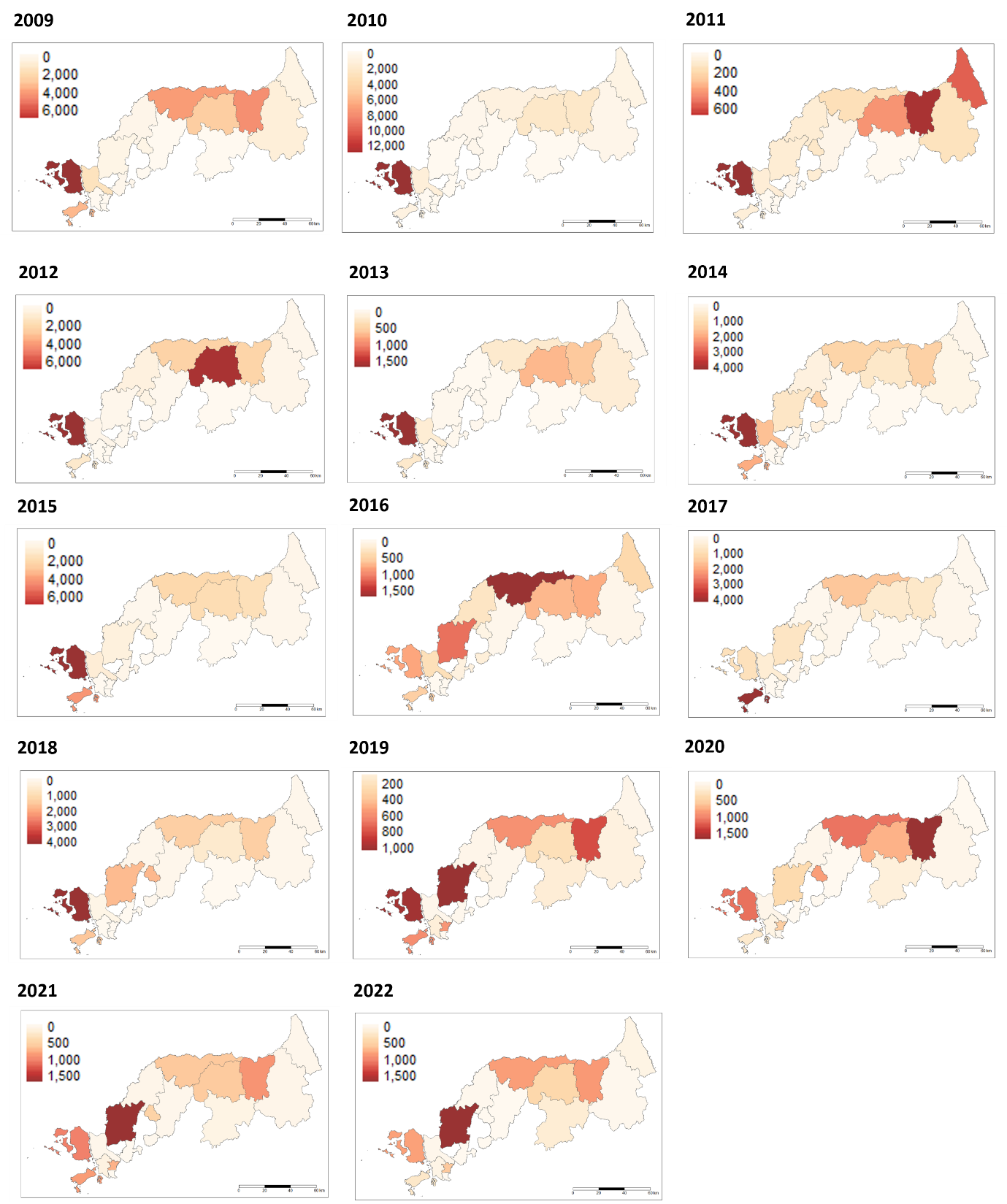
**

**Figure S7. The distribution of annual abundance of vector mosquitos in the study area from 2009 to 2022.**


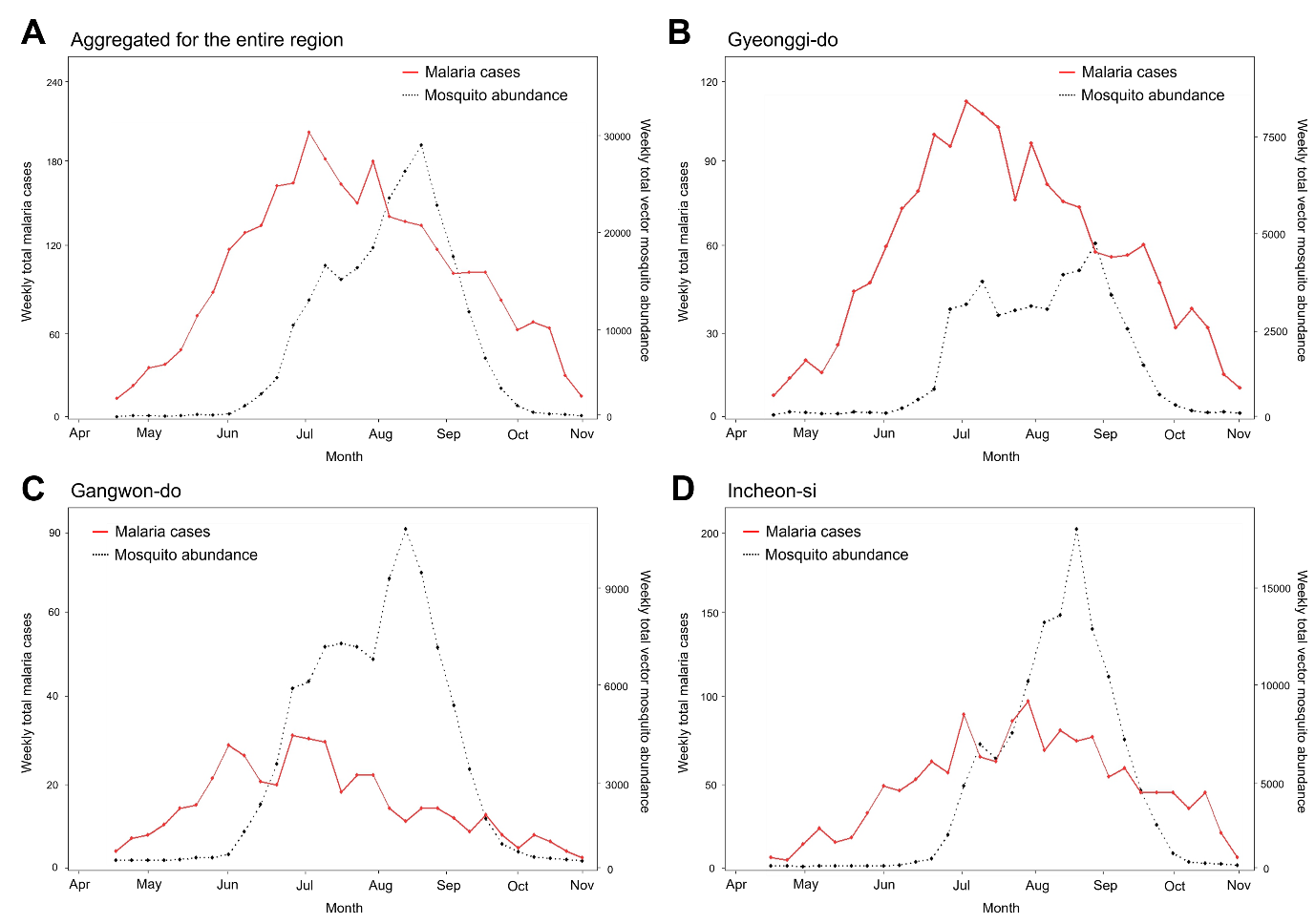


**Figure S8. Monthly aggregated malaria cases and vector mosquito abundances from 2009 to 2022.** (A) represents the results of the aggregated data for the entire study area, while (B), (C), and (D) represent the results for Gyeonggi-do, Gangwon-do, and Incheon-si, respectively.


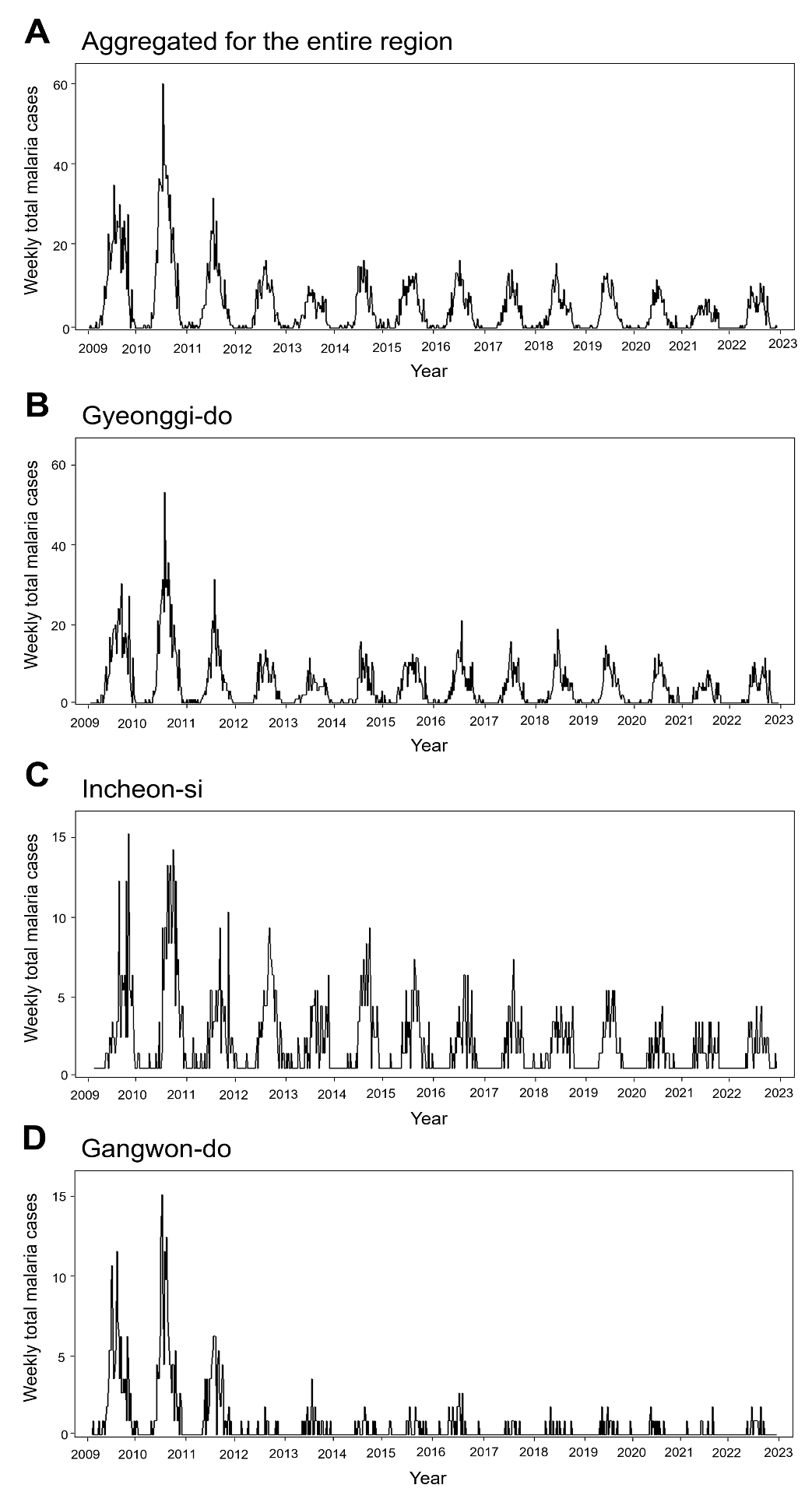


**Figure S9. Weekly total malaria cases from 2009 to 2022.** (A) represents the results of the aggregated data for the entire study area, while B, C, and D represent the results for Gyeonggi-do, Incheon-si, and Gangwon-do, respectively.


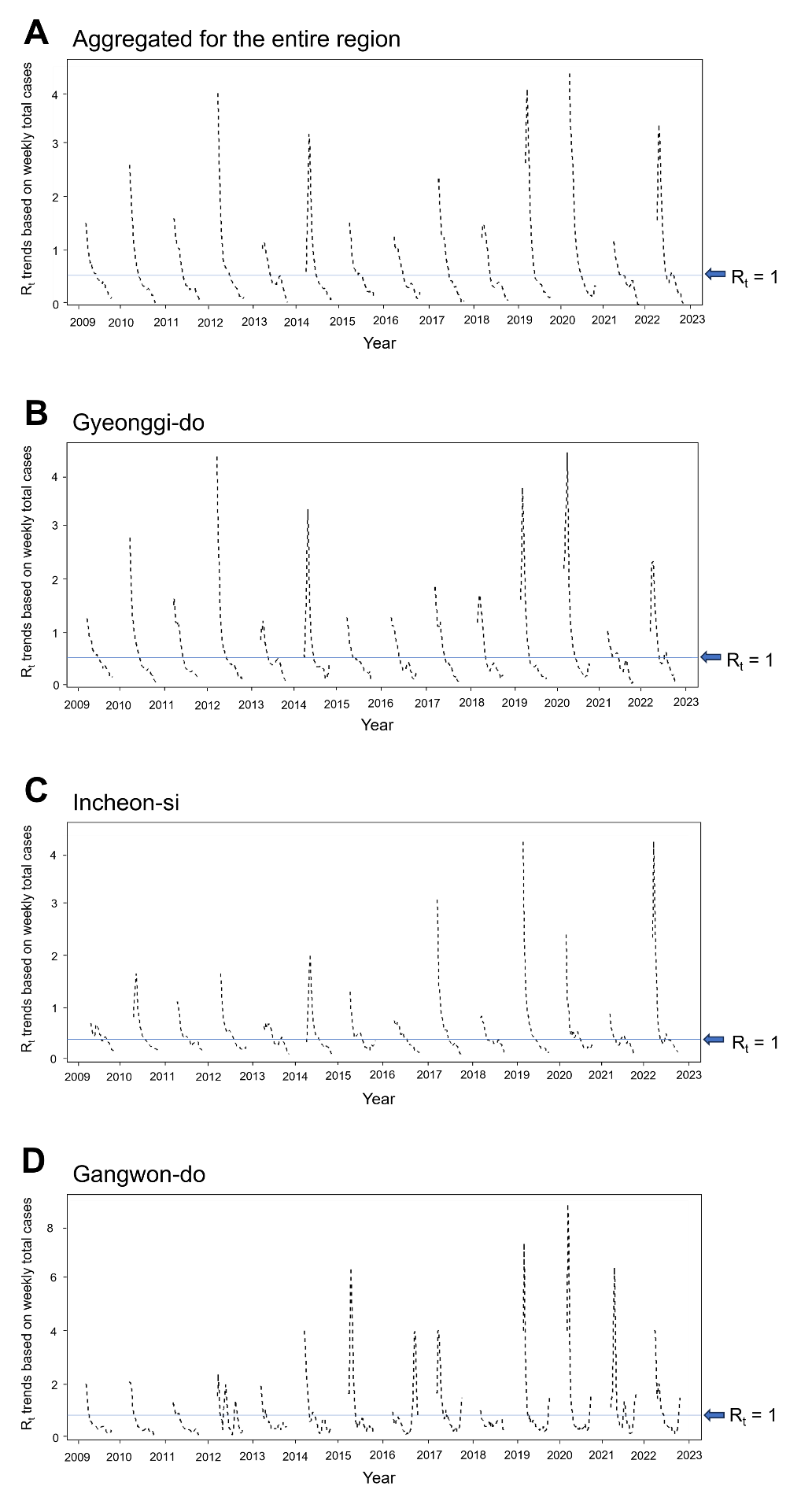


**Figure S10. Calculated R_t_ based on weekly total malaria cases from 2009 to 2022.** (A) represents the results of the aggregated data for the entire study area, while (B), (C), and (D) represent the results for Gyeonggi-do, Incheon-si, and Gangwon-do, respectively.


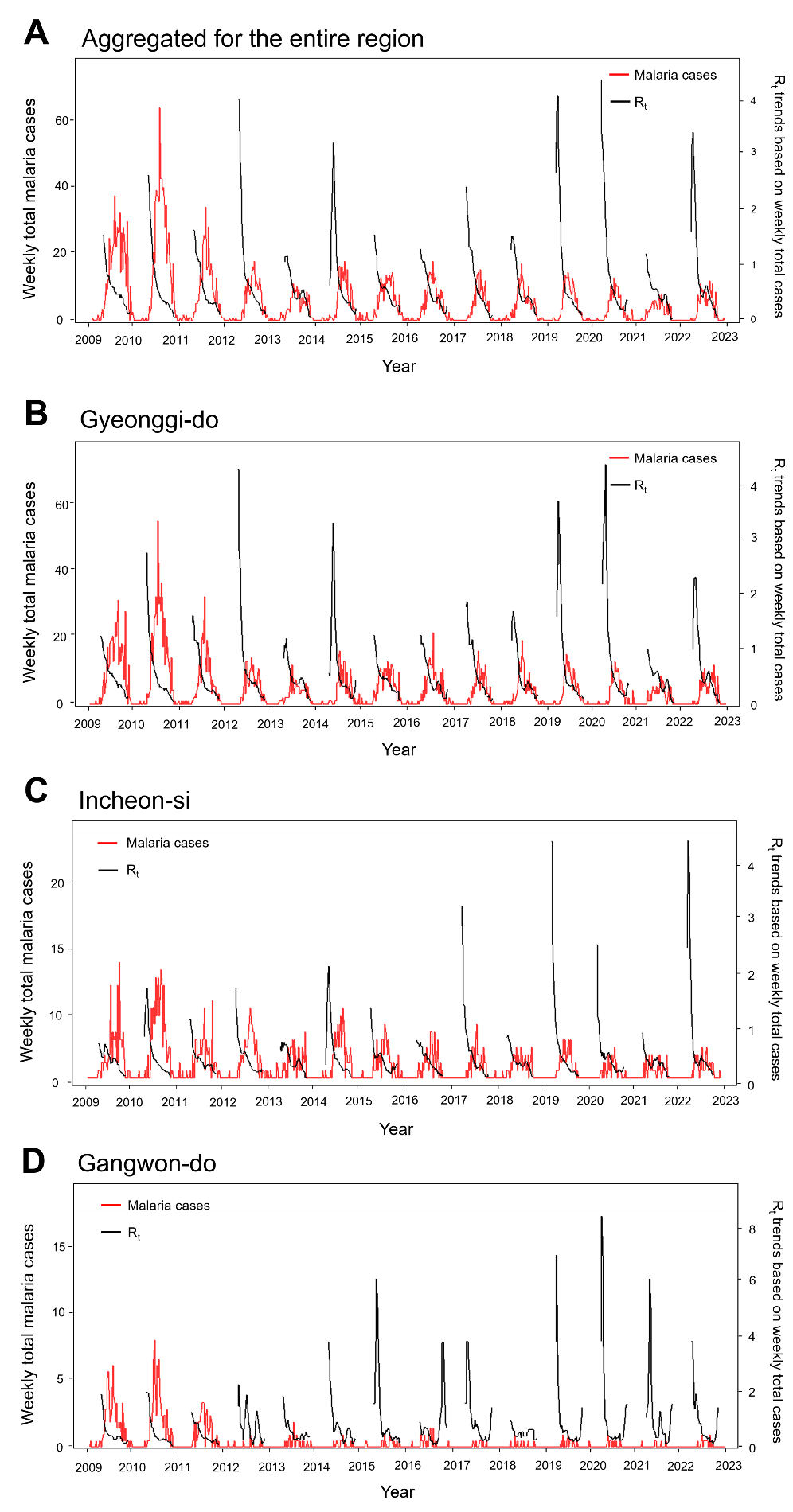


**Figure S11. Weekly total malaria cases and R_t_ based on weekly total malaria cases from 2009 to 2022.** (A) represents the results of the aggregated data for the entire study area, while (B), (C), and (D) represent the results for Gyeonggi-do, Incheon-si, and Gangwon-do, respectively.


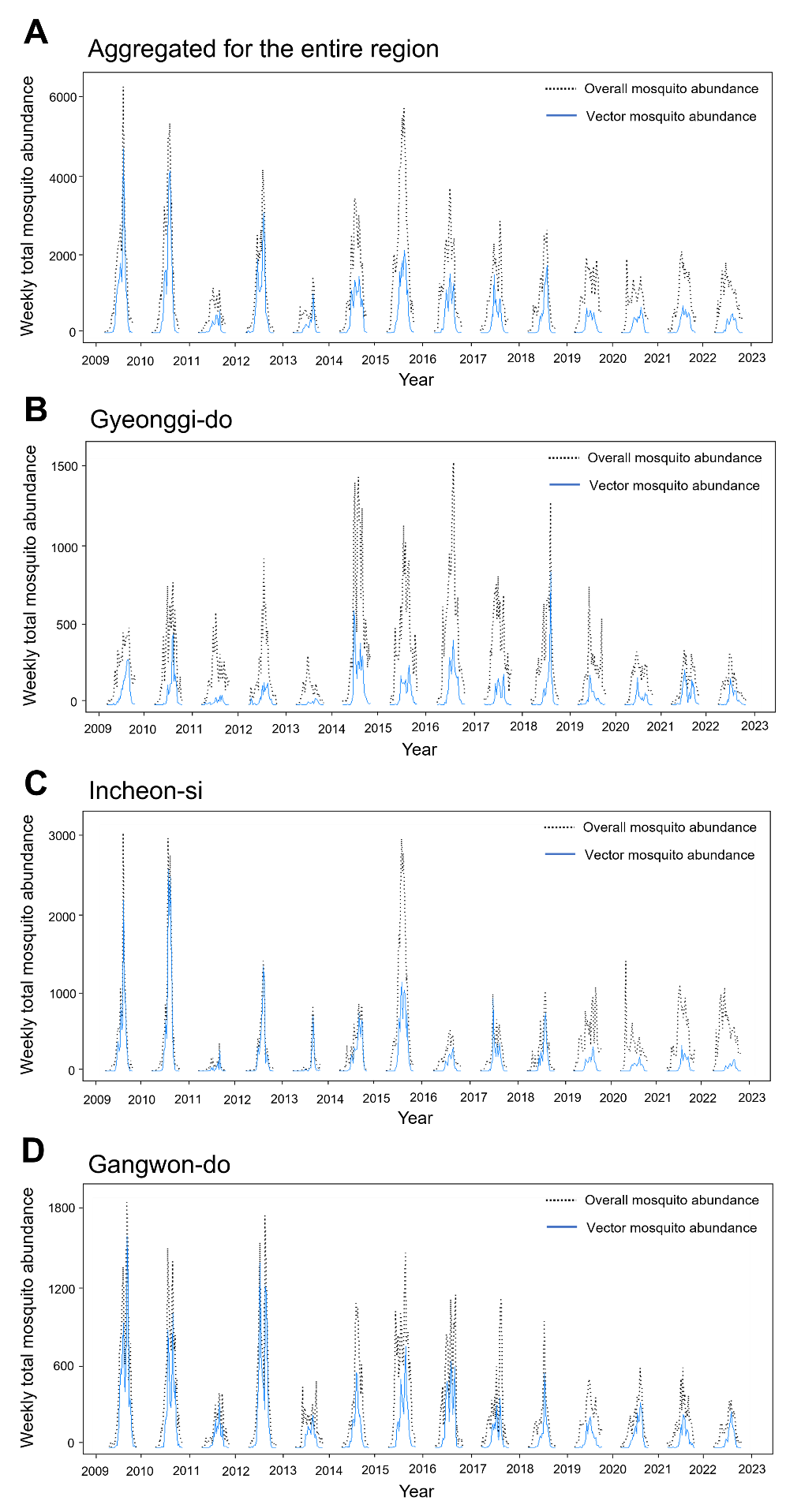


**Figure S12. Weekly total overall and vector mosquito abundance from 2009 to 2022.** (A) represents the results of the aggregated data for the entire study area, while (B), (C), and (D) represent the results for Gyeonggi-do, Incheon-si, and Gangwon-do, respectively.


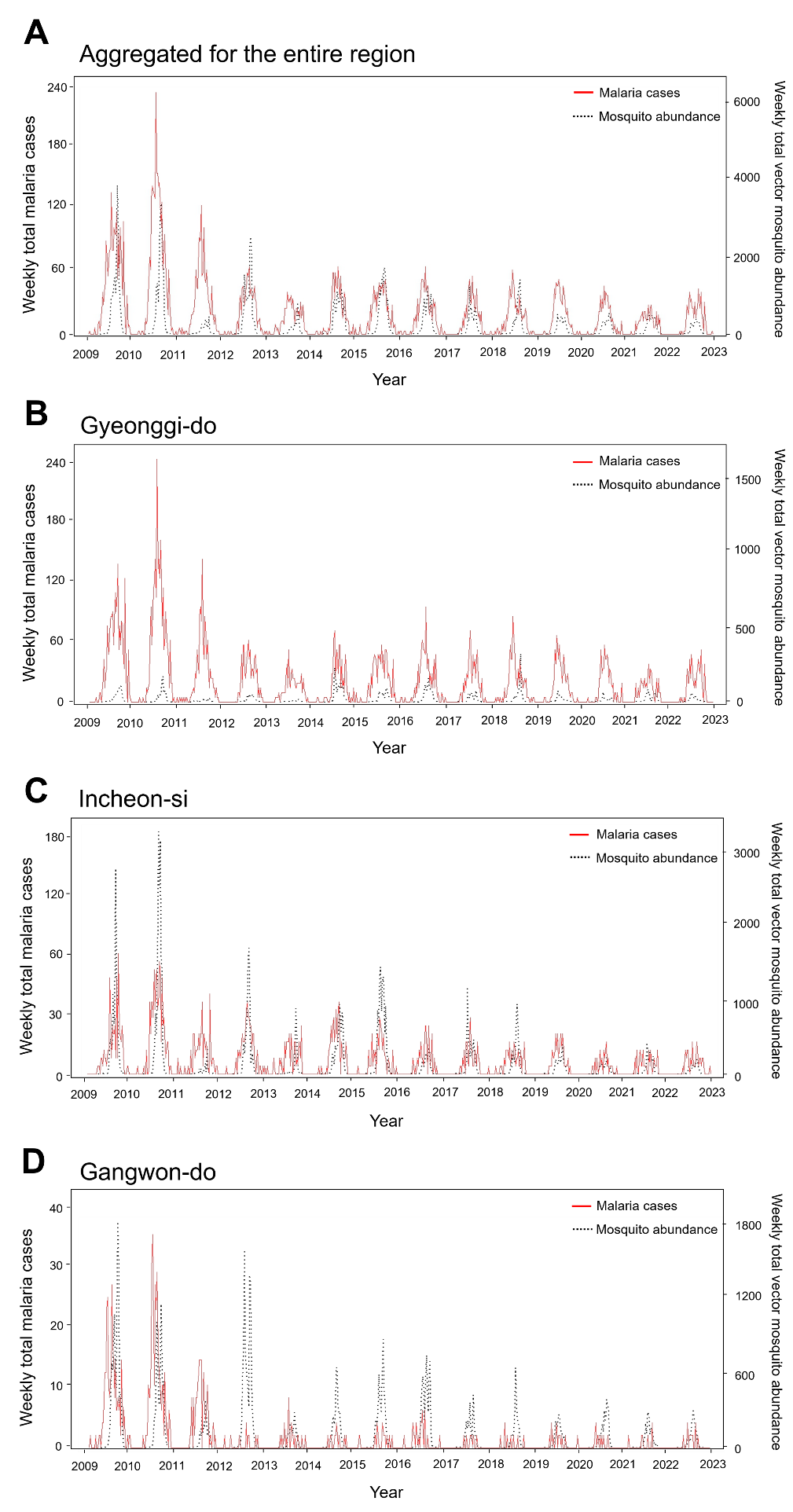


**Figure S13. Weekly total malaria cases and vector mosquito abundance from 2009 to 2022.** (A) represents the results of the aggregated data for the entire study area, while (B), (C), and (D) represent the results for Gyeonggi-do, Incheon-si, and Gangwon-do, respectively.


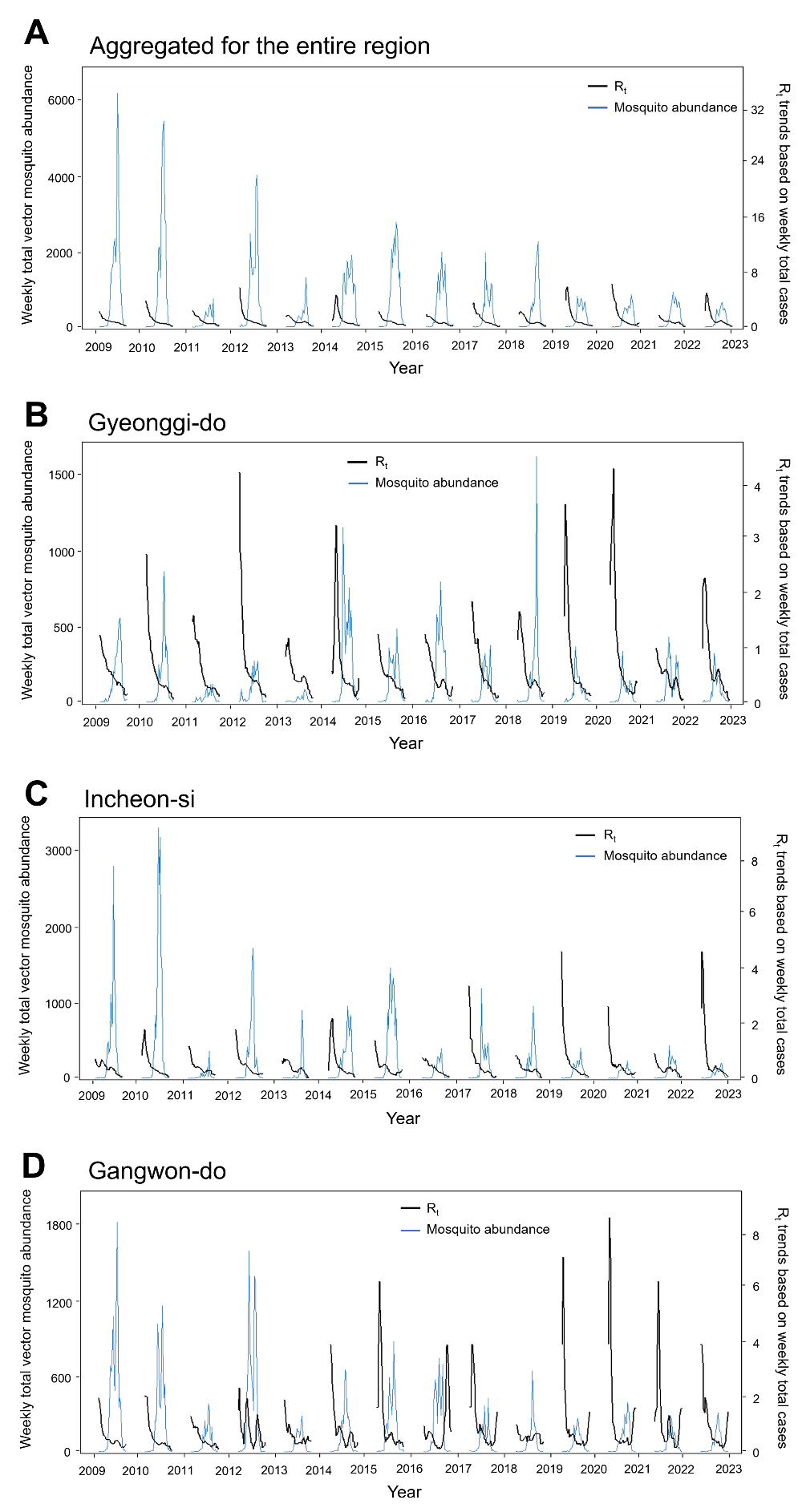


**Figure S14. Calculated R_t_ based on weekly total malaria cases and weekly total vector mosquito abundance from 2009 to 2022.** (A) represents the results of the aggregated data for the entire study area, while (B), (C), and (D) represent the results for Gyeonggi-do, Incheon-si, and Gangwon-do, respectively.


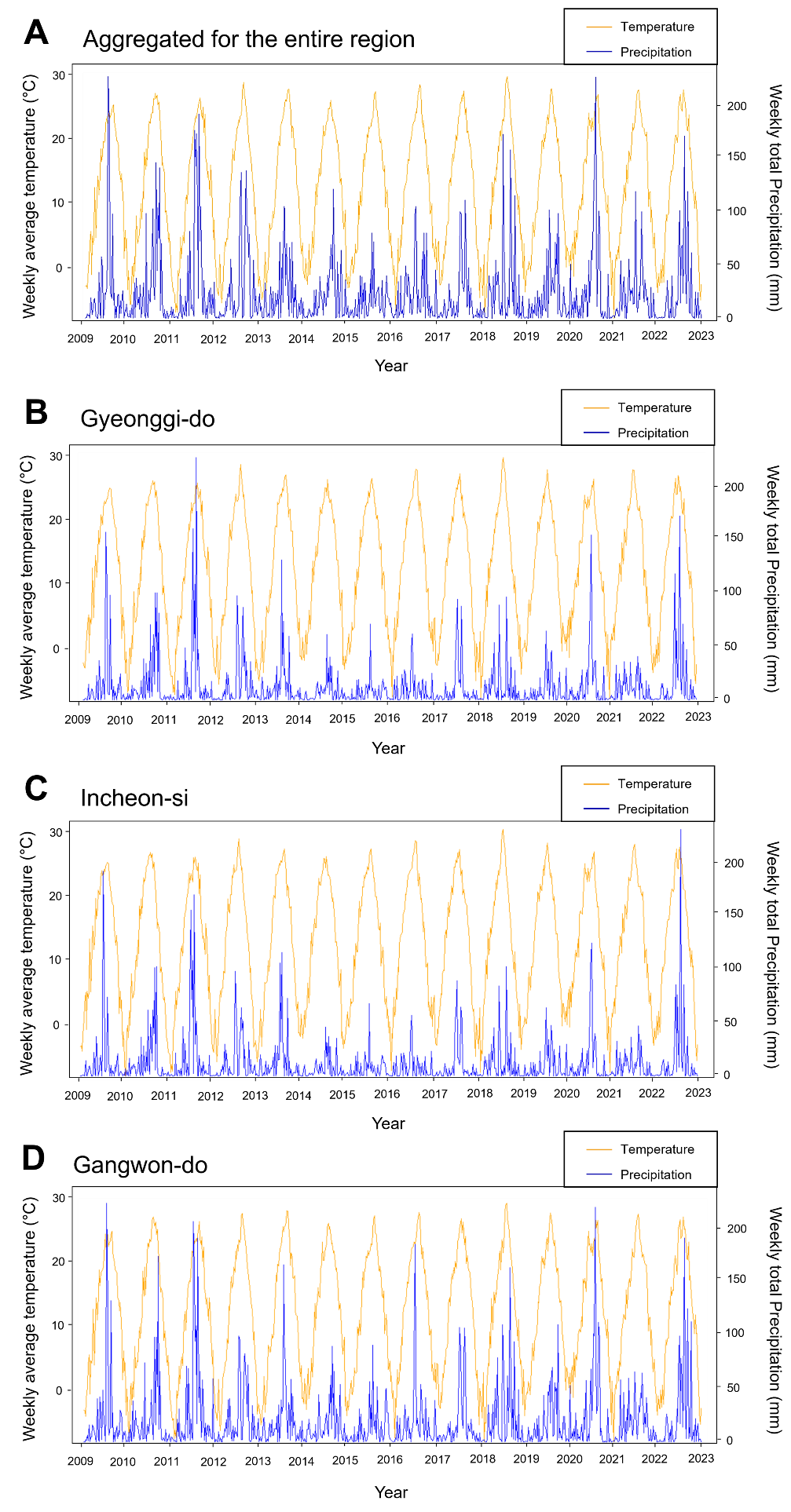


**Figure S15. Weekly average ambient temperature and weekly total precipitation from 2009 to 2022.** (A) represents the results of the aggregated data for the entire study area, while (B), (C), and (D) represent the results for Gyeonggi-do, Incheon-si, and Gangwon-do, respectively.


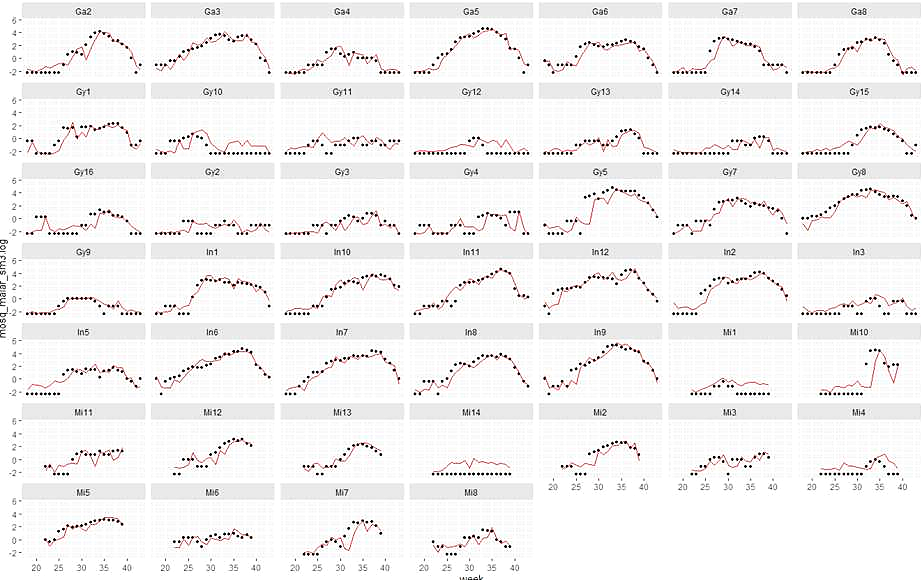


**Figure S16. Visualization of predicted and observed values of vector mosquito abundance based on GBMs.** Variable set 1 and mosquito abundance from two weeks prior were used, with a 3-week moving average applied. Results were presented for each mosquito collection site, which are the prediction units of the machine learning model. Black dots represent observed values of vector mosquito abundance and red lines display nowcasted values of vector mosquito abundance.


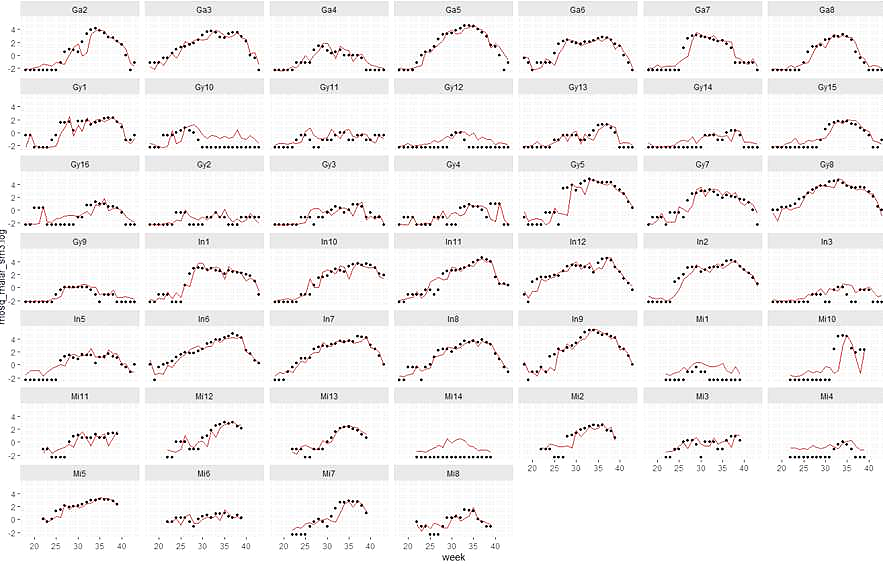


**Figure S17. Visualization of predicted and observed values of vector mosquito abundance based on XGBs.** Variable set 1 and mosquito abundance from two weeks prior were used, with a 3-week moving average applied. Results were presented for each mosquito collection site, which are the prediction units of the machine learning model. Black dots represent observed values of vector mosquito abundance and red lines display nowcasted values of vector mosquito abundance.


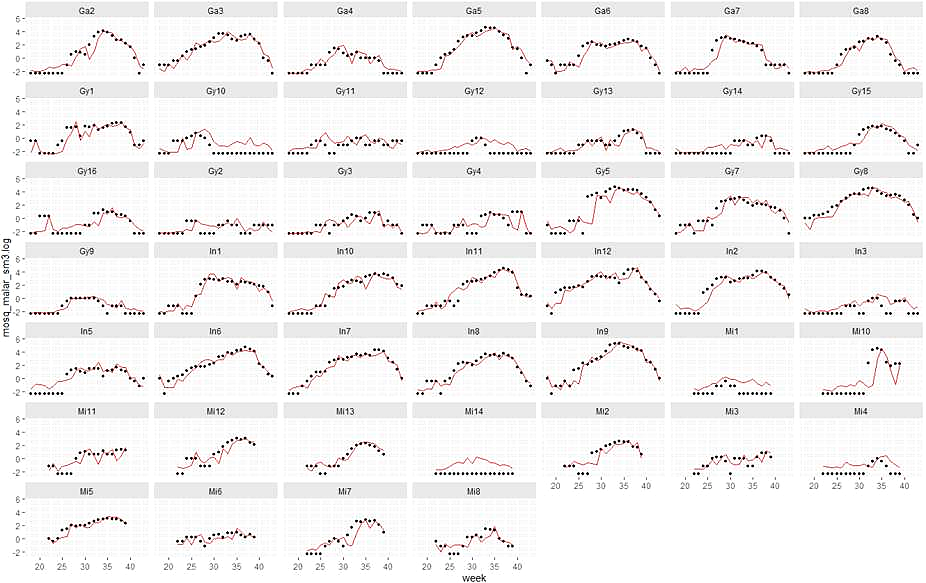


**Figure S18. Visualization of predicted and observed values of vector mosquito abundance based on ensemble models.** Variable set 1 and mosquito abundance from two weeks prior were used, with a 3-week moving average applied. Results were presented for each mosquito collection site, which are the prediction units of the machine learning model. Black dots represent observed values of vector mosquito abundance and red lines display nowcasted values of vector mosquito abundance.
